# Effect of Estrous Cycle on Behavior of Females in Rodent Tests of Anxiety

**DOI:** 10.3389/fpsyt.2021.711065

**Published:** 2021-08-31

**Authors:** Thelma A. Lovick, Hélio Zangrossi

**Affiliations:** ^1^Physiology, Pharmacology & Neuroscience, University of Bristol, Bristol, United Kingdom; ^2^Department of Pharmacology, Ribeirão Preto Medical School of the University of São Paulo, Ribeirão Preto, Brazil

**Keywords:** anxiety, female, animal models, estrous cycle, conflict, unconditioned fear, conditioned fear, sex differences

## Abstract

Anxiety disorders are more prevalent in women than in men. In women the menstrual cycle introduces another variable; indeed, some conditions e.g., premenstrual syndrome, are menstrual cycle specific. Animal models of fear and anxiety, which form the basis for research into drug treatments, have been developed almost exclusively, using males. There remains a paucity of work using females and the available literature presents a confusing picture. One confound is the estrous cycle in females, which some authors consider, but many do not. Importantly, there are no accepted standardized criteria for defining cycle phase, which is important given the rapidly changing hormonal profile during the 4-day cycle of rodents. Moreover, since many behavioral tests that involve a learning component or that consider extinction of a previously acquired association require several days to complete; the outcome may depend on the phase of the cycle on the days of training as well as on test days. In this article we consider responsiveness of females compared to males in a number of commonly used behavioral tests of anxiety and fear that were developed in male rodents. We conclude that females perform in a qualitatively similar manner to males in most tests although there may be sex and strain differences in sensitivity. Tests based on unconditioned threatening stimuli are significantly influenced by estrous cycle phase with animals displaying increased responsiveness in the late diestrus phase of the cycle (similar to the premenstrual phase in women). Tests that utilize conditioned fear paradigms, which involve a learning component appear to be less impacted by the estrous cycle although sex and cycle-related differences in responding can still be detected. Ethologically-relevant tests appear to have more translational value in females. However, even when sex differences in behavior are not detected, the same outward behavioral response may be mediated by different brain mechanisms. In order to progress basic research in the field of female psychiatry and psychopharmacology, there is a pressing need to validate and standardize experimental protocols for using female animal models of anxiety-related states.

## Introduction

It is well-established that the prevalence of psychiatric disorders encompassing anxiety-related pathologies is much higher in women than in men (1–3). Women are also likely to experience more adverse reactions to some psychoactive drugs than men (4). The menstrual cycle is another significant influence on psychiatric pathology. For example, a perimenstrual exacerbation of symptoms has been reported in women diagnosed with a psychotic disorder; admissions to psychiatric hospital are more common during the peri-menstrual relative to non-peri-menstrual phase of the cycle (5). Some anxiety-related disease states in women are menstrual cycle specific e.g., premenstrual syndrome/premenstrual dysphoric disorder, or feature a worsening of symptoms in the premenstrual phase e.g., panic disorder (6).

Given the clinical finding, it is perhaps surprising that animal models of fear and anxiety, which form the basis for research into drug treatments for humans, have been developed almost exclusively, using males. The sex bias in neuroscience and biomedical research is startling. A survey in 2007 comparing studies of behavioral pharmacology using rats and mice published in 5 reputable journals revealed that more than 80% used only male models (7). Ten years later the situation had hardly changed (7) despite the requirement by NIH and an increasing number of grant awarding bodies worldwide for consideration of sex differences in research proposals (8). Although the situation is now improving, there still remains a paucity of work using females and the available literature on sex differences presents a confusing picture.

Much of the reticence toward working on female animal models stems from the perceived difficulties and variability introduced by the cyclical variation of female sex hormones during the estrous cycle. Since steroid hormone molecules are generally lipophilic, they pass readily through the blood-brain barrier so that the female brain functions in a constantly changing chemical milieu. It should be mentioned that sex hormones may also impact on male behavior, although this is invariably neglected in studies using males for screening of drugs or to unveil the neural/chemical bases of psychopathalogies. Testosterone for example, which influences male dominance, also has a positive effect on the emission of stress-induced 22-kHz calls (9).

To overcome the source of hormonal variability in females, one strategy has been to ovariectomise animals, thereby stabilizing hormone levels. Against a stable baseline, exogenous hormones can be added back in a controlled manner to study their effects on brain circuitry and behavior. This approach has merit in that it has revealed much important information about the cellular actions of different neuroactive steroid hormones at genomic (nuclear) and non-genomic (membrane) levels (10), as well as the impact of artificial manipulation of hormones on behavior. But by its very nature, neutering removes the essence of what it is to be female. The plummeting hormone levels following the procedure may trigger adverse behavioral changes. Ovariectomy can precipitate anxiety- and depressive-like behaviors in female rats (11, 12). Indeed, in young, i.e., pre-menopausal women, hormone replacement therapy is offered following surgical hysterectomy and/or oophorectomy, precisely to prevent the development of adverse emotional states and cognitive decline (13).

There is mounting evidence that responsiveness to drugs with anxiolytic effects, including alcohol, can vary during the estrous cycle (14–19). An understanding of the changes in brain neurochemistry during the estrous cycle is therefore fundamental to the development of targeted pharmacological treatments for women. Consideration of estrous cycle-linked effects on behavior must not be overlooked and should be incorporated into the design of female animal models of psychiatric pathologies.

Progress toward this goal is however, dependent on the availability of models that are sensitive to estrous cycle stage. At present the literature presents a confusing picture. Choice of behavioral test and strain of rat or mouse as well as differences in housing environment and experimental protocols are likely only some of the sources of variability between laboratories. The lack of universally accepted criteria for staging the estrous cycle undoubtedly introduces another significant source of variability.

Clinical practice in psychiatry and psychobiology builds on the use of appropriately relevant and robust animal models (20–22), never more so than in relation to development of sex-specific pharmacology for treatment of affective disorders in women. In this short review we highlight a number of commonly used tests of anxiety- and fear-related behaviors that were developed and validated in males. We consider the limited information available on how females behave in these tests, whether there are sex differences in responding and in particular, whether the estrous cycle influences responding in females.

### The Estrous Cycle

**Figure 1:**
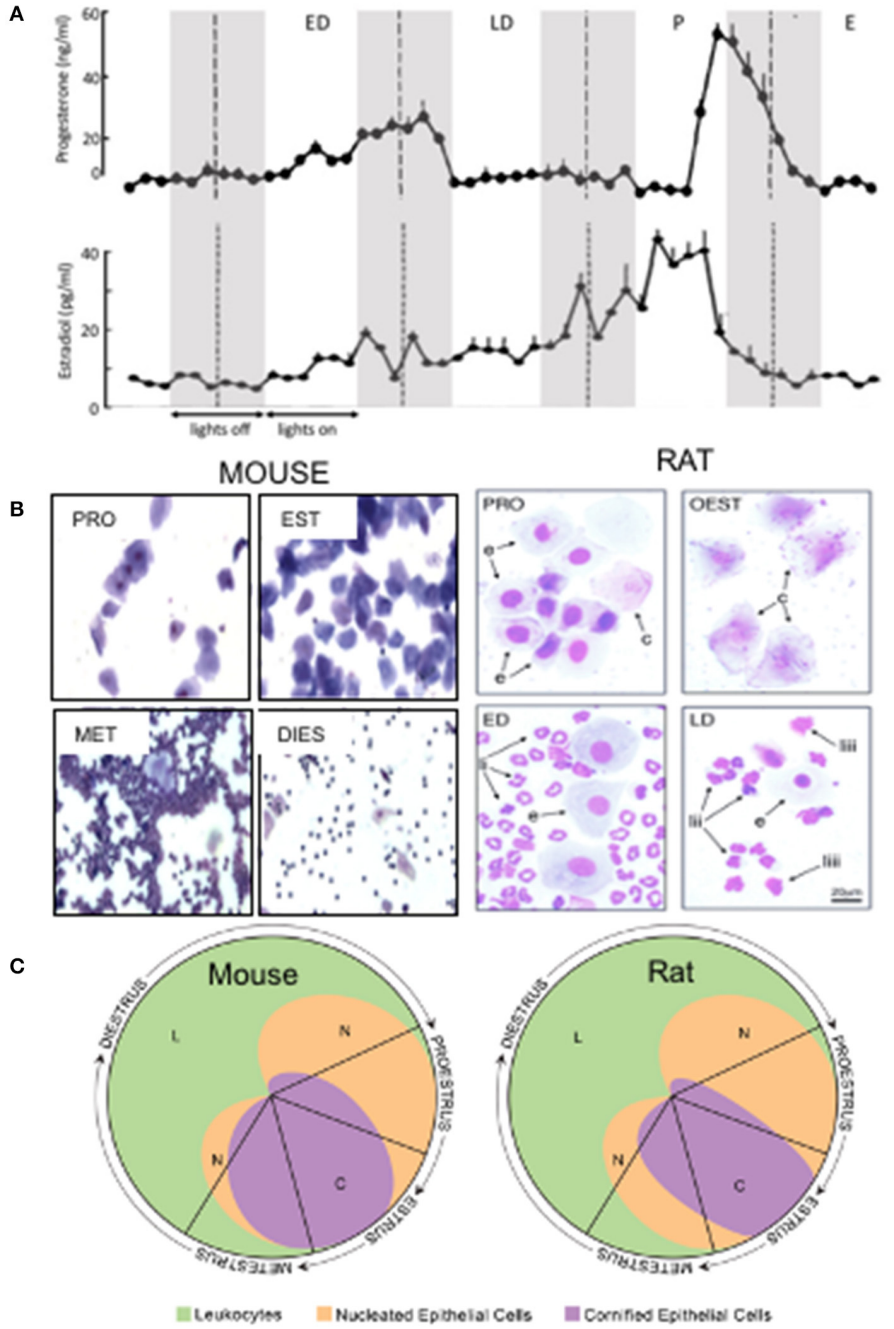
**(A)** Plasma concentration of estradiol and progesterone during the 4 day estrous cycle in Sprague Dawley rats. Shaded panels indicate dark period; broken lines indicate midnight. Approximations of cycle stages are marked by broken lines set arbitrarily at midnight. P, proestrus; E, estrous; ED, early diestrus (diestrus I); LD, late diestrus (diestrus II). Adapted from Smith and coworkers (24). **(B)** Photomicrographs show the characteristic cytology of vaginal smears obtained from rats (25) and mice (26). In rats the cycle stages were classified as proestrus (PRO), estrus (OEST or EST), early diestrus (ED) and late diestrus (LD) whilst in mice the authors subdivided the diestrus stage into metestrus (MET) and diestrus (DIST). Showing round-nucleated epithelial cells (e), larger, cornified cells (c) and polymorphonuclear leucocytes with distinctly lobed nuclei (li) or clumped nucleus (liii). Note magnification of mouse smears is lower than for rats but scale bar not available for mice. **(C)** Relative proportions of different cell types in vaginal smears at different stages of the estrous cycle in rat and mouse. From Cora et al. (27) adapted from the Byers and Taft (28) estrous cycle identification tool.

The estrous cycle in rats and mice, the most commonly used species for behavioral research, is characterized by a four or sometimes 5 day long cyclic variation in secretion of ovarian hormones. The duration of the cycle may be less consistent in mice, varying from 2 to 8 days (23). During this time the two major sex hormones: estrogen (17β estradiol in rodents) and progesterone undergo dramatic out-of-phase fluctuations in the level of secretion (Figure 1A). Since these lipophilic steroid molecules pass readily through the blood brain barrier, their concentration in the plasma is followed by parallel changes in concentration in the brain where both hormones are neuroactive, acting on genomic (nuclear) as well as at membrane-bound receptors, the latter leading to rapid non-genomic effects on membrane excitability (29).

There are no universally accepted criteria for defining cycle stage and much confusion arises in the behavioral literature with respect to estrous cycle stages. Classification of estrous cycle stage is undoubtedly one of the major factors that contributes to variability and lack of reproducibility of results obtained by different laboratories using rodents to model anxiety-related disorders in women. For behavioral experiments, sampling plasma hormone levels is not practicable as a routine procedure to assess gonadal status. However, changes in peripheral hormone levels are reflected by a changing vaginal cytology as the reproductive tract is primed to prepare for pregnancy. In non-mated, single sex-housed females, the cycle can be subdivided into a number of stages based on vaginal cytology. This provides a convenient, if imprecise, surrogate for the changing hormonal profile within the brain.

Samples for cytological evaluation are obtained from the vaginal wall, either by lavage (flushing the vagina with water or buffer) or by inserting a probe into the vagina to obtain a cell sample. Three types of cell can readily be distinguished: lymphocytes, keratinised squamous cells and leucocytes, the proportions of which vary throughout the cycle. The reader is referred to excellent descriptions and illustrations of cell types (30, 31). During the cycle, four stages of unequal duration: proestrus, estrus and diestrus, the latter commonly subdivided into two stages, may be identified according to the appearance and relative number of different cell types, with a gradual rather than step-like transition between stages. For convenience, most laboratories conduct behavioral experiments during the animal's light period, having collected a vaginal smear in the morning of that day. Typically, in rats proestrus and estrus are assigned a day each, starting at midnight whilst diestrus, which lasts longer, is subdivided into two periods termed by different workers early and late diestrus, diestrus I and II or metestrus and diestrus (Figure 1A).

### Vaginal Cytology and Cycle Stage

***Proestrus*** is readily identified by the presence of nucleated epithelial cells, which in smears stained with Giemsa or similar stains, purple nuclei are clearly observed within a blue cytoplasm (Figures 1B,C). Proestrus typically lasts around 14 h but within that time widow there are rapid changes in the hormonal profile. In rats maintained on a 12 h on 12 h off light-dark cycle (lights on at 06.00 h) used in many laboratories, progesterone secretion remains very low from midnight (00.00 h on the day of proestrus) until about 15.00 h in the afternoon when a rapid spike in secretion starts. Progesterone concentration peaks in the evening in the early part of the dark phase, then declines rapidly to basal level by around midnight (00.00 h) (24, 32). Estradiol, which has been rising gradually over the previous 3 days, reaches peak concentration at around midday (12.00 h), and then declines, returning to basal level by the late afternoon (24, 32). Thus, mornings are characterized by low progesterone and high estradiol whereas during the afternoon the surge in secretion of progesterone leads to the highest concentration achieved during the cycle, whilst estradiol concentration is declining rapidly (24, 32). Given the rapidly changing hormonal profile during proestrus, the timing of behavioral experiments during the day of proestrus deserves consideration as a potential source of variability.

In estrus, which lasts 24–48 h the nucleated lymphocytes characterizing proestrus are replaced by large, keratinised squamous cells (Figures 1B,C). Secretion of progesterone and estradiol remain at a low stable level throughout estrus (24, 32).

Diestrus is the longest lasting phase and the source of the most discrepancies in classification. The diestrus period is characterized by an abundance of leucocytes in smears (Figures 1B,C) but the number, appearance and presence of other cell types and of mucus in the smears varies. As mentioned above, most workers subdivide diestrus into two phases variously termed metestrus and diestrus; diestrus I and II, or early and late diestrus. These terms are not necessarily interchangeable and precise cytological descriptions of the criteria applied for classification are essential although regrettably, not available in all studies. At the beginning of diestrus progesterone secretion begins a progressive rise, which continues until the early morning of the second day when secretion terminates abruptly, precipitating a rapid fall in concentration (Figure 1A). In contrast, estradiol remains relatively stable during this period (Figure 1A). Rapid withdrawal from progesterone has been shown to trigger plasticity of GABA_A_ receptor subtype expression that leads to significant changes in excitability of brain circuits associated with anxiety (33–35).

#### The Estrous Cycle—Mice

Increasingly, mice are being used for behavioral research in order to capitalize on the availability of an increasing number of genetically modified strains, which can help in defining the neurochemistry of emotional behavior. Female mice display similar, but not identical, changes in vaginal cytology to rats during their estrous cycle (Figures 1B,C). However, it is much more difficult to imply a causative link between changing levels of brain neuroactive steroid hormones and behavior in mice compared to rats. Diurnal fluctuations in adrenal secretion of progesterone and its metabolites, which undergo a surge in the dark period, far outweigh ovarian secretion rates (36). Moreover, the level of adrenal secretion of progesterone also appears to be estrous cycle stage dependent (37), unlike in the rat (38). Metabolism of progesterone in mouse brain also differs from the rat (39) but perhaps most importantly, the concentrations of progesterone and its metabolite tetrahydroprogesterone (TH PROG) in female mouse brain are caused predominantly by changes in the supply of endogenous brain progesterone (36), rather than peripheral sources.

### Refinements and Alternatives to Vaginal Cytology

There is a pressing need to standardize classification of estrous cycle stages to facilitate comparisons between cycle-linked changes in behavior reported by different laboratories. Several attempts have been made to develop rapid, objective methods, particularly for use in mice. These include using non-stained smear material (27, 40), modifications to staining methods (41) and application of deep learning technology for classification of smears (42). Alternatives to vaginal cytology have been proposed based on gross examination of the vaginal opening (43), changes in skin temperature due to activation of brown adipose tissue (44) and variations in vaginal wall impedence (45). To date, none of the latter methods has been widely adopted and vaginal cytology remains the gold standard for assessing estrous cycle stage. Avoidance of handling stress is also an important source of variability that needs to be considered. Vaginal lavage can lead to raised plasma corticosterone with associated deficits in spatial memory (46). In male rats, even the acute stress of handling in animals not habituated to the procedure, leads to raised brain concentration of progesterone (47). A similar effect may be produced in females. In skilled hands however, smear collection is minimally stressful and the stained smears provide a permanent record available for objective scrutiny by blinded personnel. Even so, classification of the diestrus stage remains a source of confusion between studies. For the purposes of simplicity, with the caveat that criteria for defining these stages may differ between laboratories, in this article we will use diestrus I to also include the phases termed by different authors metestrus, early diestrus and dietrus 1. Diestrus II encompasses stages termed late diestrus or diestrus 2. Diestrus refers to studies in which no distinction has been made between stages. There is clearly a pressing need for a universally agreed consensus on cytological criteria for estrous cycle staging to facilitate comparison between results obtained from different laboratories.

## Animal Models of Anxiety—Behavior of Females in Male Models

The emotional states of fear and anxiety have an adaptive value. Novel stimuli/situations present a potential threat to the survival of an individual. In small prey species the most pragmatic response is usually to escape or alternatively, to become immobile in order to reduce the risk of being detected. This strategy becomes maladaptive if it is applied indiscriminately in response to all novel stimuli since other behaviors essential for survival such as foraging or finding a mate, will be compromised. Instead, the animal needs to display a level of vigilance in order to detect changes in its environment and then to assess the level of threat and the risk before deciding whether a modification to its ongoing behavior is appropriate (48).

Behavioral tests in animals that are aimed to elicit fear and anxiety are broadly based on two tenets: (i) fear operates to move the animal away from danger. It involves fight/flight/freezing, and these defensive responses are more commonly evoked by unambiguous, immediate/proximal threats, such as the confrontation with a predator (49); (ii) anxiety refers to a preparatory response to possible future threatening events, especially in situations where there is conflict between different goals, such as between avoiding a potential threat and being attracted to food (50). It can be translated in behaviors such as risk-assessment, including the scanning of the environment and hyper-attentiveness to the potential threat, along with disruption of ongoing behaviors (51).

Animal models of anxiety and fear can be broadly grouped into two main subclasses: the first involves ethologically based paradigms based on an animal's spontaneous or natural (unconditioned) reactions (e.g., flight, avoidance, freezing, risk-assessment) to stress stimuli that do not explicitly involve pain or discomfort but represent a threat to survival (e.g., exposure to a novel highly illuminated test chamber or to a predator). The second includes animals' conditioned responses established following exposure to stressful and often painful events (eg, electric footshock) (52, 53).

Below, we consider a number of behavioral tests that were developed in males, and the available information regarding the behavior of females in these tests. The findings are summarized in Table 1.

**Table 1:** Sex and estrous cycle effects on fear/anxiety in commonly used behavioral tests in rats and mice.

**Test**	**Species**	**Sex differences**			**Estrous cycle**	
		**M>F**	**F>M**	**M=F**	**No effect**	**Diest>Pro/Est**
**Unconditioned threat**						
Elevated plus maze	Rat	61–63			9,63, 73–5	18,40,66–72
	Mouse			64,65	23	68,70,76-8
**Elevated T—maze**						
Inhibitory avoidance	Rat	97		94,95,96	96	94
	Mouse					
Escape	Rat			94–96		
	Mouse					
Open field	Rat	62,101–104		60,63	107,109	68,109
	Mouse			105	32,108	32,70
Light-dark test	Rat			118-124		18,145–6
	Mouse					
**22kH USV**						
Mild restraint	Rat	154				154
	Mouse					
Air puff	Rat	9,156		154		
	Mouse					
Predator	Rat	150				
	Mouse					
Acute hypoxia	Rat					178a
	Mouse					
Prey-Predator	Rat	184	149,183	185		186
	Mouse					
**Conditioned threat**						
Vogel conflict	Rat		61,194–6		194	
	Mouse		197			
Conditioned fear	Rat		211		154,204–5, 213	
	Mouse					
Fear potentiated startle	Rat	200–3	209	154,204–7		
	Mouse			208	107–8	212
Fear extinction	Rat		210			206,212–3
	Mouse			215		

### Tests Based on Unconditioned Threatening Stimuli

#### Elevated Mazes

##### Elevated Plus-Maze

**Figure 2:**
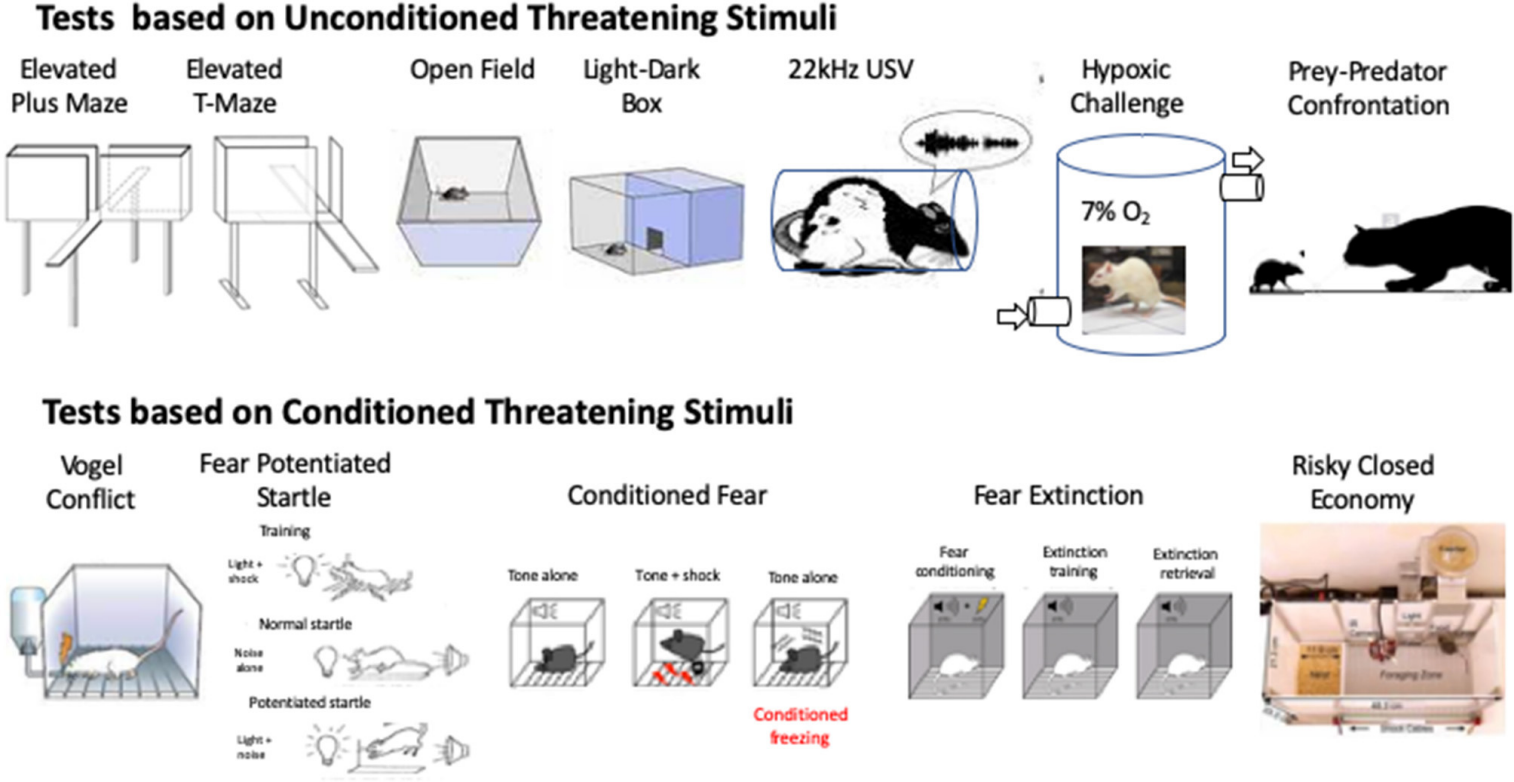
Schematic diagrams to illustrate tests based on unconditioned threatening stimuli (top row) and conditioned threatening stimuli (Bottom row).

First proposed by Handley and Mithani (54) and further validated by Pellow et al. (55), the elevated plus maze (EPM) exploits the natural tendency of rodents to explore novel environments and their innate avoidance of unprotected and elevated places (Figure 2). When placed in the center of the maze, the number of times the animal visits the open arms and the time spent there reflect the choice the animal makes when balancing the putative reward offered by exploring its new environment (entering the open arm) weighed against the potential threat of danger posed by the new environment.

The EPM is the most widely-used animal model for investigating the pathophysiological bases of anxiety, as well as for screening anxiety-modulating drugs and mouse genotypes. After nearly 40 years of use, with nearly 8700 publications listed as of May 2021 (PubMed, NIH National Library of Medicine), the EPM remains the gold standard against which other behavioral tests for anxiety are measured, at least in males (52, 56, 57). Even so, some have expressed reservations about the use of the EPM (20).

Despite its widespread use, relatively few studies have considered sex differences in responding, or the effect of the estrous cycle in females. The consensus based on the limited literature available, is that adult female rats behave in a qualitatively similar manner to males in the EPM but display overall lower levels of anxiety (58–61). Sex differences were not however, observed in young adult mice (C57BL/6NIA strain) although interestingly, aged females were found to be more anxious than males (62, 63).

Once the estrous cycle is taken into account, the picture starts to become less clear. In rats, some investigators report reduced anxiety levels (i.e., a more time spent in the open arms of the maze) in proestrus/estrus compared to diestrus (16–18, 40, 64–70) whereas others fail to see estrous cycle-linked effects at all (16, 19, 61, 71–73). However, not all workers compare all stages of the cycle and in some cases results from two stages of the cycle have been pooled, which makes direct comparison between studies difficult.

The results from studies in mice paint a similarly confusing picture. Some studies report that mice in proestrus show more open arm entries and spend a longer time on the open arm of the EPM than diestrus females or males (66, 68, 74, 75) whilst others found that mice in estrus spent longer on the open arm than mice in diestrus (76) and yet others were unable to detect any difference in performance between mice in estrus and diestrus I (defined as receptive stages) and diestrus II and proestrus (defined as non-receptive stages) (23).

In males, many factors have been identified that can influence responding and potentially lead to inconsistencies between results from different laboratories due to methodological differences. The importance of recognizing and carefully controlling testing conditions, particularly light level, has been highlighted (73, 77, 78). The age of the rat, circadian phase and light illumination level during testing have all been reported to influence the behavior of males (77, 79–84) but see (85) for an opposing view.

Responsiveness of females has not been subjected to the same level of scrutiny. There is however, some evidence that strain (particularly in mice), light level and circadian phase may affect responsiveness (26, 65, 86).

In terms of assessing influence of the estrous cycle, the difficulties in making comparisons between results obtained from different laboratories are further compounded by the fact that in many cases, only two stages of the cycle have been compared, typically proestrus and diestrus (not subdivided into the substages), whilst in other studies data from two phases is pooled e.g., proestrus/estrus, thereby precluding evaluation of the effect of an individual cycle stage. At present there is no consensus.

##### Elevated T-Maze

The EPM, discussed above, may be viewed as a mixed model of anxiety/panic, because it combines two defensive strategies: (1) inhibitory avoidance, when the animal is in the enclosed arms and refrains from entering an open arm and (2) one-way escape, when the animal retreats from one of the open arms to seek the enclosed arm. The unstable balance between the expression of these two types of responses could explain the inconsistencies of drug effects in males, mainly 5-HT-modulating compounds, frequently reported for the EPM (87–89). To circumvent the ambiguities of the EPM Graeff and coworkers (90, 91) developed the elevated T-maze (ETM). The ETM differs from the EPM by sealing the entrance to one of the enclosed arms (Figure 2). As a result, it consists of three arms of equal dimension, one enclosed and two open, all elevated above the floor.

The test allows the measurement in the same rat, of an approach-avoidance conflict-type response: inhibitory avoidance, which is related to anxiety, and an escape response which is related to fear/panic. When placed at the end of the enclosed arm, the rat cannot see the open arms until its head is poked beyond the walls of the enclosed arm. Because the open arms are aversive, the animal will learn inhibitory avoidance when repeatedly placed at the end of the enclosed arm and allowed to explore the maze. On the other hand, when the rat is placed at the end of one of the open arms it can move toward the safer enclosed arm, performing an escape response, termed one-way escape, which is associated with fear/panic attacks. Contrary to what happens in the enclosed arm, the latency to leave the open arm usually does not change with successive trials. Anxiolytic drugs (e.g., diazepam and buspirone) impair inhibitory avoidance acquisition, while leaving escape expression unaltered. Antipanic drugs such as the antidepressants fluoxetine or imipramine, or high potency benzodiazepines (e.g., alprazolam and clonazepam) inhibit escape expression [for a full account of the test see (92)].

The limited comparisons made between male and female rats in this test show no sex-differences for inhibitory avoidance acquisition or escape expression in Wistar rats (93–95). However, female Long-Evans or Sprague-Dawley rats show a deficit in avoidance learning compared to males, indicating a less anxious phenotype (96). Therefore, it seems that performance in the test may vary between different strains of rat.

With regard to the influence of estrous cycle phases, one study reported that female Wistar rats in diestrus II are slightly more reactive to the open-arms than males (i.e., they take longer to leave the enclosed arm but only in the first trial), indicating a higher anxiety level (93). However, this subtle effect was not replicated in a recent study with the same rat strain (95).

#### Open Field Test

Individual laboratory-based anxiety tests, which by their nature are artificial, probably reflect different facets of emotionality (97) and viewed in isolation, cannot provide a complete picture of an animal's emotional profile. To overcome this limitation, it has been proposed that using a battery of tests may provide a more reliable measure, at least in males (98, 99).

Behavior in an open field or more correctly, a walled arena (Figure 2), is often paired with other tests and used as a measure of locomotor activity as well as anxiety. The former is typified as the total distance traveled in a given time, whilst time to re-enter the center of the arena, or time spent in the central portion of the arena is used as a surrogate for anxiety, reflecting the choice made by the animal as it pits the novelty of exploring a new environment against the risk of danger posed by leaving a safe area next to the walls.

Overall, female rats have been reported to be more active in the open field compared to males, showing greater ambulatory and rearing activity and defecating less than males (100–103) and appearing to be less anxious about entering the central zone. However, the literature is far from consistent, with several examples of no difference in behavior between sexes. In mice too, a retrospective analysis of performance in the open field concluded the performance of female and male mice was equivalent (104). In male mice strain differences have been reported in locomotor activity in the open field as well as anxiety using the EPM (97, 105); sex differences in responding are evident within some strains although not others (97).

Pooling data from all females risks masking possible effects of estrous cycle stage on responding. Even so, when estrous cycle has been taken into account, the findings are equivocal. For example, rats in proestrus showed greater anxiolytic behavior than at other stages (66). Conversely, no differences were detected in anxiety-like or fear behaviors between proestrus and diestrus rats (106). Similarly in mice, some workers report proestrus wildtype mice BALB/cBy to make more entries and spend longer time in the central zone of the open field compared to their diestrus (stage not subdivided) counterparts (26, 68) whilst others using C57BL/6J mice found that behavior in the open field remained stable across 4 phases of cycle (26, 107).

A recent insightful study assessing behavior in the open field in rats has demonstrated how multiple variables can acutely modulate each other in different contexts, and highlights the importance of considering each of these factors. Miller et al. (108) observed independent interactions between the estrous cycle and novelty (experiencing the open field for the first time), estrous cycle and light, and novelty and light wherein each factor is concurrently influencing behavior. Novelty was found to obscure estrous cycle effects. Similarly estrous cycle-linked effects were not evident in experiments carried our under white light, which rats find aversive, but could be observed in experiments conducted under dim red lighting (108). Another factor that appears to influence responding is the size of the field. Female rats in a large open field (129 × 120 × 60 cm, dim red light 18lux) spent more time in the central zone and made more central zone entries than males (60). However, using smaller arenas [70 × 70 × 70 cm arena, light level not specified (58) and 54.5 × 80 × 33 cm arena, dim red light 18lux] (61) no sex difference was observed in either distance traveled or entries into the central zone. These apparently conflicting findings are a concern. However, given the limited visual acuity of laboratory rats, large arenas may be perceived to pose more of a threatening challenge than small ones. The higher level of exploratory behavior displayed by females compared to males in large arenas may therefore reflect a real sex difference in terms of intrinsic level of anxiety.

When the open field test is incorporated into a battery of tests designed to assess fear and anxiety, conflicting findings have been reported regarding sex differences and estrous cycle-linked effects on anxiety-like behavior in the same animals exposed to the EPM and to the large open field (59, 61, 97, 102, 109–113). Importantly, an estrous cycle-related influence on behavior in the open field does not necessarily predict the behavior of the same animal in the EPM (58, 60, 61, 68, 114).

The aforementioned studies present a confusing picture. Given the numerous factors that can influence responding in the open field and the EPM, it is likely that methodological differences between laboratories are major factors that contribute to the lack of consensus regarding sex differences or the effect of the estrous cycle. What is evident is that females behave in a qualitatively similar manner to males in both the open field and elevated plus maze tests but whether there are sex differences in responding remains an open question. In the studies that have found sex differences, females have generally displayed lower anxiety levels compared to males. This finding is in complete contrast to humans in whom the incidence of anxiety-related pathological states is not only higher in women compared to men but also the symptoms experienced by women are often menstrual cycle-related.

#### Light-Dark Transition Test

The light-dark transition model was developed by Crawley and Goodwin (115, 116), based on the exploratory behavior of rodents in a two-compartment box, where one chamber is brightly lit and the other dark (Figure 2). In such conditions, mice and rats have a clear preference for the dark side of the box and the number of transitions made by them between the two compartments and the time spent in the brightly lit side have most commonly been used as indices of anxiety.

Although a reasonable number of studies in the literature has compared the behavior of males and females in these tests, very few have explored the impact of the cycle phases on the female response. The majority of the studies performed either with rats (117–123) or mice (63, 124–130) have failed to show sex-differences in this test. In some of these studies direct comparisons among strains (97, 131, 132) and/or age of the animals (133, 134), which are critically-relevant variables, were performed but no sex-related effect was found. There are however, a few reports showing that females are more (135–138) or less (139–143) anxious than males.

Regarding the cycle phases, female rats in proestrus or in estrus+proestrus are less anxious compared to the other phases (18, 144, 145), or with males (144, 146). In the only study available in mice a lower anxiety level was detected in the proestrus and estrous phases compared to diestrus or to males (114). It is clear that pooling data from females can mask significant sex differences due to the influence of a changing hormonal profile during the estrous cycle.

#### 22 kHz Ultrasonic Vocalizations

Rodents use a range of ultrasonic calls to communicate the presence of positive or negative emotional states and to coordinate social interactions (147). In male rats in a semi-naturalistic environment (visible burrow system) the presence of a predator (domestic cat) stimulates animals to emit high frequency ultrasonic vocalizations (USV) at around 22 kHz (148). The 22-kHz USVs are thought to act as a warning to conspecifics since far fewer calls are made if the confrontation with the predator occurs when the rats are remote from their social group (149, 150). Female rats also emit 22 kHz USVs but their calls are longer and more frequent than those made by males (149, 151). Interestingly mice, which are also prey for cats, do not emit such cries in similar threatening situations (150). Adult mice do communicate utilizing USVs but at other frequencies, primarily in the context of social interaction (152).

In rats in the laboratory setting a number of stimuli have been identified that evoke innate defensive escape behaviors that include emission of 22 kHz USVs. These include mild restraint stress (153) (Figure 2); air puff (154, 155); forced swimming (156); overhead looming stimuli simulating aerial attack (157) and unavoidable acute or repeated footshocks (158). The 22 kHz USVs emitted in these settings are widely believed to reflect a negative affective state akin to anxiety and fear (159).

Compared to the extensive literature on 22 kHz USVs made by male rats in laboratory-based tests, USVs in females remain largely overlooked. The limited available information suggests that females may be less responsive than males. In response to air puff stress females emit fewer 22 kHz calls than male rats although interestingly, freezing evoked by the same stimulus did not differ between sexes (9, 155). Female Wistar rats submitted to a short period non-noxious restraint stress also emitted far fewer 22 kHz calls than male rats (153). However, within the female cohort used in this study there was a marked effect of estrous cycle stage. Females in their proestrus, estrus and early diestrus (diestrus I) phases emitted very few calls, but during the late diestrus stage (comparable to diestrus II) calls increased 5-fold, reaching a level comparable to males (153). In the air puff test Inagaki and Mori (9) also failed to detect differences between the 22 kHz USVs emitted by rats in proestrus and diestrus I. However, since responsiveness in other stages of the cycle was not investigated, it is not possible to conclude whether the estrous cycle impacted on this test. These findings do however contrast with reports that female Long Evans rats living in a semi-naturalistic environment (the visible burrow system) made more frequent cries in the presence of a predator than males (149). Whether this reflects a strain difference, or an influence of the living environment is not clear.

#### CO_2_ and Hypoxia Challenges

A wealth of evidence shows that respiratory challenges such as exposure to a high concentration of CO_2_ or a low concentration of O_2_ evoke panic attacks in humans (160–162); these stimuli have frequently been used as experimental tools to study panic disorder (161, 163). Although the pathophysiological mechanisms of panic disorder remain unclear, there is compelling evidence linking this psychiatric condition to respiratory disturbances [for a review see (164)].

The use of respiratory challenges to model panic attacks in experimental animals has been less straightforward, and the results obtained raise doubts that a panic-like state was indeed evoked in these non-human subjects. Broadly speaking, in these analyses, conducted mostly in male rats and mice, different parameters, primarily autonomic indexes (i.e., arterial blood pressure and heart and respiratory rates) have been used to infer that an extreme fear response, and hence a panic-like state, was evoked (165–169). Investigation of the behavioral consequences induced by CO_2_ inhalation or hypoxia has also been carried out in some cases but curiously, this has habitually been done after, and not during, exposure to the respiratory challenges [e.g., (170–174)]. Efforts have been made to investigate whether changes in these cardio-respiratory indices are sex-dependent, or influenced by the estrous cycle, but their results, as recently reviewed (175), have not been conclusive.

Our laboratory reported that Wistar male rats submitted to acute hypoxia (7% O_2_) display a panic-like escape response (i.e., upward jumps to the border of the experimental cage) (Figure 2), which is reduced by treatment with standard panicolytic drugs such as fluoxetine and alprazolam (176). We also observed that these drugs are equally effective in reducing the number of escape attempts made by mice during exposure to a high CO_2_ concentration (20%) (177), validating these two behaviorally-oriented tests for the study of panic-attacks in male rodents.

Recently, we have also validated the hypoxia model for use in females. We observed that exposure to 7% O_2_ evokes panic-like escape behavior in both male and in female Sprague Dawley rats. However, in females, reactivity to this respiratory challenge was clearly dependent on the stage of the estrous cycle, being significantly higher in diestrus II, compared to other cycle stages or to males (178). This finding has an important translational value since women with panic disorder experience an increase in anxiety and panic symptoms during the premenstrual phase of the menstrual cycle (179, 180), which corresponds to diestrus II in rodents.

#### Predator-Prey Interaction

Exposure to predators or stimuli related to them (e.g., predator odor) has been widely used to assess defensive behavior in rodents. The influential ethoexperimental studies conducted by Caroline and Robert Blanchard have long guided research in this field. Through the use of two ingenious test batteries, the Fear/Defense Test Battery (F/DTB) and Anxiety/Defense Test Battery (A/DTB) (149, 150, 181, 182), these researchers addressed the pattern of defensive behavior expressed by male and female rodents exposed to these naturalistic threats. While the former battery has given information on the defensive behaviors displayed by rats to present, approaching predators (a live cat) (Figure 2), such as flight/escape, freezing and defensive attack, the latter investigates reactions to potential threat (e.g., cat odor), such as risk-assessment behaviors.

Overall, their results have shown that females are more defensive than males when confronted by these stimuli, and this is particularly common in situations involving potential, as opposed to actual and present, threat (148, 183). Females, for instance, display more risk-assessment and avoidance behaviors than males do in response to cat odor (148).

It is noteworthy, however, that conflicting results have also been reported by other groups. Perrot-Sinal et al. (184) observed that exposure to cat odor increased the expression of risk-assessment behaviors in both male and female rats, but with a significantly lower frequency in females. However, when animals were submitted to chronic restraint stress prior to testing, females displayed a higher incidence of these behaviors than males. This indicates that the anxiety/stress basal state of the animals before the test can influence the way they respond to predatory stimuli. On the other hand, exposure of rats to an odor stressor of a different predator (trimethyl thiazoline; the main component of fox feces) increased, in a sex-independent manner, the expression of defensive behaviors, such as risk-assessment activities and defensive burying (185). Interestingly, the similarity in behavioral responsiveness masked sexually dimorphic changes in cell proliferation and death in the hippocampal dentate gyrus (185).

More recently, Pentkowski et al. (186) investigated the impact of estrous cycle phases on the unconditioned and conditioned defensive responses of female rats to cat odor. They observed that rats in diestrus 2 displayed significantly higher levels of risk assessment responses during exposure to a cloth impregnated with cat odor than in estrus or proestrus phases. When 24 h later, the animals were reintroduced to the cage where the odor was presented (now having a control cloth, without cat odor, which served as a stimulus-paired cue) in order to explore the conditioned responses to the experimental context/cue, a significant increase in the defensiveness was observed in the animals previously exposed to cat-odor (i.e., increased time spent in risk-assessment activities and avoiding the cue), demonstrating aversive learning. In contrast to the initial exposure (unconditioned response), there was no influence of the cycle phases on the learned response.

Finally, it is noteworthy that besides the effects of predator odors on defensive behaviors, it has been shown that exposure of weanling female rats to cat odor, for 10 consecutive days, interferes with the maturation of the hypothalamic-pituitary gonadal axis, leading to a delayed vaginal opening and first estrus, besides disrupting estrous cyclicity (187).

### Tests Based on Conditioned Threatening Stimuli

#### Vogel Conflict Test

The Vogel test is based on the approach-avoidance conflict generated in rodents between an appetitive drive: to drink water after a period of water-deprivation, and the fear of doing so as water consumption is punished by electric shocks delivered either to the animal's paws or tongue (Figure 2). Since its introduction in 1971 (188), this test has been widely used for the screening of anxiolytic drugs and to unveil the pathophysiological bases of anxiety (189–193). As mostly inferred from studies with males, anxiolytic drugs, such as the benzodiazepines diazepam and chlordiazepoxide, consistently increase the number of punished responses (190, 194).

As with other models, few studies have directly compared the behavior of male and females in this test. Overall, female rats (59, 194–196) and mice (197) exhibit a reduced number of punished responses compared to males, suggesting a higher anxiety level. However, this conclusion has been questioned by evidence showing that female rats may have increased sensitivity to pain, a lower shock threshold perception and exhibit reduced unpunished drinking responses compared to males. The latter effect, which indicates a different baseline water intake, was observed after controlling for body weight of both sexes, an important and normally overlooked confounding variable [for a review of these findings see (194)].

To date, only one study has addressed the impact of the cycle phase on female behavior. Basso et al. (194) failed to find any significant difference between cycle phases in the number of punished responses exhibited by adult Wistar female rats. They also reported that the cycle phases had no impact on the effects of the anxiolytic drugs tested in their study.

#### Conditioned Fear and Fear Potentiated Startle

Two commonly used indices of fear responses in male animals are based on the association of specific stimuli (cued or contextual) with stressful and often painful events (e.g., electric footshock) (52, 53). In conditioned fear responses (CF) (198), rats are trained to associate a conditioned stimulus (CS), (typically light or sound) with an aversive unconditioned stimulus (US; footshock). The animals are then re-exposed to the CS alone in a different context. Freezing in response to the CS is then taken as an index of conditioned fear (Figure 2). Fear potentiated startle (FPS) is a related test that measures the potentiating effect on the startle response to a loud sound, of presentation of a CS that has previously been paired with an aversive US (footshock) (199) (Figure 2). As a whole, female rats perform in a qualitatively similar manner to males in both tests and, although some workers find females less responsive than males (200–203), others have failed to detect sex differences (107, 153, 204–207).

Similarly, inconsistent findings have been reported in mice. For example, depending on strain and precise experimental protocol, no sex difference in contextual fear conditioning (208); stronger context fear conditioning and more generalization to a similar context have been reported in females compared to males (209), whilst extinction of conditioned freezing to a tone was faster in males than in females (210). Using a serial compound conditioned stimulus (tone and white noise that elicits clear transitions between freezing and flight behaviors within individual subjects) females exhibited more freezing behavior than males although there was no difference between the sexes in flight behavior (211).

When estrous cycle phase has been considered, the consensus from the limited number of available studies is that it does not influence expression of fear-potentiated startle (106, 107) although a more recent study presents a conflicting view (212), nor does it impact on conditioned fear to context (153, 204, 205, 213). In contrast, in tests of cued fear (conditioned freezing) Milad et al. (206) found that extinction training during the proestrus phase (high estrogen/progesterone) was more fully consolidated, as evidenced by low freezing during a recall test. Others reported weaker extinction during training in rats in diestrus II (212), or in the diestrus phase [not subdivided (213)] compared to proestrus i.e., rats continued to respond to presentations of the unreinforced CS for longer during the test session compared to rats in proestrus in which the response extinguished rapidly.

A consideration when using tests that involve a learning element, is that they take several days to complete and typically involve two or more sessions. This means that females may be conditioned in one stage of their estrous cycle but tested on another day, when they are in a different stage. There is a possibility that cycle stage during conditioning (or during the test session) may impact on responsiveness during the test session, and *vice versa*. In the limited studies that have addressed this question, estrous cycle phase seems not to impact significantly on the training or testing components in the conditioned fear paradigm (204, 212). On the other hand, gonadal status does affect fear potentiated startle. Females tested in proestrus after conditioning in proestrus or diestrus II of the previous cycle, appeared initially to fail to distinguish between a positive and neutral conditioned stimulus, although performance improved as the test session progressed (212). Circulating estrogen levels are high in proestrus and estradiol has been shown to promote fear generalization to context (214–216). An apparent failure in discriminatory learning of rats tested during proestrus, may in fact reflect generalization to positive and negative conditioned stimuli, rather than a failure of learning (212). In another recent study employing startle, but in a fear safety conditioning paradigm, female rats in diestrus I or II had significantly reduced safety memory compared to females in the proestrus or estrus phase (217).

It is interesting to speculate why some tests of conditioned fear are affected by estrous cycle stage and not others. Fear potentiated startle differs from other commonly used tests of fear by measuring enhancement rather than suppression of ongoing behavior (199). It may be that this factor renders the test more sensitive to the effects of hormonal fluctuations in females. The above examples do, however, emphasize not only the importance of drug testing in both males and females, but also the choice of behavioral test. In addition, they highlight that neglecting the influence of the estrous cycle in females may lead to erroneous interpretation of data in some behavioral tests.

#### Tests Based on Fear Extinction

In recent years much attention has been focussed on extinction of conditioned fear responses. In humans, deficits in extinction of conditioned fear during repeated presentation of an unreinforced CS are considered a contributory factor underlying anxiety disorders (218, 219). Anxious individuals show more elevated fear responding to a CS during extinction relative to healthy controls (220). Patients with posttraumatic stress disorder (PTSD) also continue to exhibit a robust conditioned fear response even after undergoing extinction training (221). In animals, fear extinction: the decrement in conditioned fear responses that occurs with repeated presentation of an unreinforced conditioned fear stimulus (Figure 2), may therefore provide a useful model to help understand the underlying psychopathology of anxiety states (222). Deficits in the extinction of fear memory and the way this impacts on subsequent interpretations of and reactivity to sensory events may be at the core of PTSD. It is worth noting however, that in classical Pavlovian terms, extinction implies gradual waning of the conditioned response as a consequence of non-reinforcement of the conditioned stimulus (CS). In PTSD, the individual does not usually experience the exact CS again. Rather, they appear to generalize to the original traumatic event so that other stimuli act as a CS and trigger an aversive reaction. Nevertheless, the use of extinction recall/retention following fear conditioning has gained currency in conditioned fear models of PTSD.

PTSD is twice as common in women than in men (223); moreover in women, menstrual cycle phase has been reported to influence extinction retention (224). The importance of incorporating females into animal tests of fear extinction cannot be overstated. In rats, the phase of the estrous cycle prior to extinction training (testing the response to the unreinforced CS 24 h after training) can influence extinction recall 24 h later. Rats that underwent extinction learning in diestrus I displayed poorer retention of extinction compared to animals undergoing extinction learning in proestrus (206).

As females are gradually incorporated into experimental protocols, it is becoming clear that even when sex differences in behavior are not detected, the same outward behavioral response may be mediated by different mechanisms. A pertinent study utilizing conditioned fear in mice reported similar levels of fear extinction in males and females (215). However, the similarity in behavior between the sexes belied differences in the underlying pharmacology. Whilst in males, extinction and subsequent renewal of fear were enhanced by administration of a presynaptic GABA_B_ receptor antagonist, females were unaffected (215). In a similar vein, although no sex differences could be distinguished in freezing recall in rats tested in a contextual fear paradigm, significant upregulation of the early gene ARC was detected in the bed nucleus of stria terminalis in males but not in females (225). Sexual dimorphism with respect to the involvement of endocannabinoid pathways in conditioned fear extinction has also been reported in rats (226).

### The Risky Closed Economy

The classical tests of fear and anxiety behavior in rodents assess specific behaviors (e.g., freezing) during brief sampling periods and in an artificial laboratory setting, providing only a “snapshot” of fear and anxiety-related behaviors. This limitation has driven a search for more ethologically relevant settings in which to study fear and anxiety-like behaviors. In the Risky Closed Economy (RCE) (227) animals live undisturbed, although individually housed, in a semi-naturalistic environment where they are free to acquire their food and water by lever-pressing in a designated foraging zone (Figure 2). An unsignaled, unpredictable threat (footshock) is introduced into the foraging zone to model the risk of predation.

Arguably, this test should afford a more holistic understanding of the effects of fear and anxiety on a day-to-day basis since data from a multitude of variables can be collected automatically and continuously over several weeks to months (228). When applied to females, the RCE also has the advantage of being able to follow the same animal at different stages of its estrous cycle. In terms of foraging behavior in the RCE, female rats were more fearful than males. Moreover, estrous phase appeared to influence risky foraging decisions, with increased risk taking associated with the proestrus phase (227). This finding is significant since unlike most of the behavioral tests employing less ethologically relevant scenarios, the increased level of fear or anxiety seen in female rats in the RCE during the diestrus phase parallels the human experience.

## Sex Differences in Responses to Psychoactive Drugs

It is becoming increasingly clear that male and female brains do not necessarily utilize the same neural mechanisms to achieve the same behavioral output. Moreover, as females are gradually introduced into drug testing protocols, evidence is accumulating showing sex differences in drug responsiveness as well as a differential responsiveness within females depending on the stage of the estrous cycle. Sex differences in responding to the classical anxiolytic benzodiazepines have been recognized for many years. Females are generally considered to be less responsive than males. However, the results must be viewed with caution. For example, the absence of female responses to the effects of diazepam in the EPM was found to be due to the high baseline activity levels seen with females, rather than a differential response to the drug (229). Such findings highlight the need to consider sex differences in baseline behaviors to allow for unambiguous extrapolation of results. In addition, sex and strain differences in metabolism of benzodiazepines have been reported, which can also bias results (230) although no sex difference in brain concentration was found, at least in Long Evans rats (231).

The estrous cycle also impacts on responsiveness to benzodiazepines, although once again, findings are equivocal. Most workers fail to investigate all stages of the cycle, which leads to incomplete data sets. Some studies in rats were unable to detect any estrous cycle linked differences in responsiveness to diazepam (72, 232). However, in the EPM the overall consensus is that rats and mice are more sensitive to diazepam during the proestrus/estrus phases compared to diestrus, especially diestrus II (16, 19, 233–235). Similarly, in the light-dark transition test Rodriguez-Landa and coworkers (18) found that diazepam caused anxiolytic effects in female Wistar rats in proestrus or estrus phases, but not in their diestrus phase. This may be a consequence of higher binding of diazepam to brain membranes in proestrus compared to the other cycle stages (236).

Sex and estrous cycle-linked differences in responsiveness to other anxiolytic drugs have also been reported in other behavioral tests. Whereas, no sex difference was observed in the effect of the serotonin and noradrenaline reuptake inhibitor sibutramine in rats tested in the EPM, sibutramine impaired inhibitory avoidance (withdrawal from the enclosed arm) in the ETM in males but not females, but inhibited escape expression (latency to leave the open arm) in both sexes (95). When the estrous cycle was taken into consideration, the antipanic-like effect of the drug on escape performance was found to be absent in females in diestrus II but preserved in the other cycle phases (95).

Females and male rats also differ in their sensitivity to the effects of anxiolytic drugs in the Vogel conflict test. Whereas, an increase in punished responding was observed to be equal in both sexes after acute administration of diazepam and chlordiazepoxide, anxiolysis caused by buspirone, fluoxetine, paroxetine, or propranolol was evident only in males. Moreover, female rats seem to be more sensitive to the sedative effects of buspirone and chlordiazepoxide than males (194). In another recent study employing startle in a fear safety conditioning paradigm, female rats in diestrus I and II had significantly reduced safety memory compared to females in the proestrus or estrus phases. This difference could be reversed by intranasal application of oxytocin (217) although interestingly, oxytocin had no effect in males (217).

Sex and estrous cycle stage also impact on responsiveness to the widely used serotonin reuptake inhibitor fluoxetine. Chronic fluoxetine impaired inhibitory avoidance in a one-trial step-through task in male but not female mice (237). In rats chronic (14 day) administration reduced fear responses during extinction learning and extinction recall in female rats in diestrus I and II but not in proestrus/estrus females or in males (15).

Chronic administration of fluoxetine is normally required for anxiolytic effects to develop. In contrast, acute administration evokes anxiogenic effects independent of sex (15, 193). However, at low doses that are subthreshold for its effects on 5-HT systems, fluoxetine can be anxiolytic in females, and this effect is dependent on estrous cycle stage. Administration of low dose fluoxetine in diestrus 2 was able to completely reverse the increase in unconditioned fear that characterizes this stage of the cycle. Thus, fluoxetine in diestrus 2 reversed the increase in restraint stress-induced ultrasonic vocalizations; hypoxia-induced escape behavior and vibration stress-induced hyperalgesia that characterize this stage of the cycle (33, 154, 178a) but had no effect when administered at other stages cycle (33). Fluoxetine also normalized the increased excitability of the panic circuitry in the periaqueductal gray matter that occurs during diestrus 2 (33) and restored responsiveness to diazepam in the EPM (19). These effects are thought to be related to the rapid steroid-stimulating properties of fluoxetine, which raises brain concentration of allopregnanolone and offsets the natural sharp decline that occurs at diestrus 2 (33, 238).

## Discussion and Conclusion

The available data indicates that females respond in a qualitatively similar way to males in the majority of behavioral tests used to assess fear and anxiety in male animals. The overall conclusion from the behavior of females in “male” models of fear and anxiety is that females show lower levels of anxiety compared to males (Table 1). Yet this finding is in direct contrast to the clinical experience where the prevalence of anxiety-linked disorders is higher in women than men. It is worth remembering however, that the commonly used animal tests model the adaptive states of fear and anxiety and not the psychopathology which characterizes human anxiety states like panic disorder, generalized anxiety disorder and post traumatic stress disorder. It may be that a lower intrinsic (baseline) level of anxiety in females compared to males is normal in rodent societies and should not be a concern when investigating the biological basis of anxiety behavior.

Since the readout of animal tests is mainly locomotor based, the overall higher level of activity in females could bias the result. However, recent careful analysis of locomotor activity in three tests of anxiety: EPM, open field and social interaction failed to detect an influence (61). It may be that instead of expressing less anxiety, female rats express different forms of anxiety-like behaviors that are not well-captured by the testing procedures that have been developed and characterized using male rodents. The readouts of many common behavioral tests developed and validated in male animals, may therefore need to be adjusted in order to assess the same emotional states in females. A case in point is the classic fear conditioning paradigm whereby animals freeze in response to a conditioned stimulus or to a context signaling footshock. Overall, males froze more than females, but a subset of females were more likely to engage in “darting” behavior (226, 239, 240), which could not be attributed to overall hyperactivity.

A caveat to analysis of female behavior in the traditional “male” animal tests should be a consideration of the extent to which the current animal models of fear and anxiety do actually model these emotions in humans (241). Indeed, it has been questioned whether current fear conditioning studies in rodents operate in the natural world (228) since unnatural tasks performed by rodents living in standard laboratory conditions may not model their behavior in the wild, where the living environment and challenges to survival are quite different. For example the impact of housing conditions on rodent brain and behavior (242) is well-established and has led to the adoption of various degrees of enrichment into laboratory housing conditions for rodents. In humans, an ethological approach to fear has been successfully incorporated into experimental research paradigms (243). Animal studies lag behind in this respect, although the use of the visible burrow system, first demonstrated 30 years ago (148), which enables observation of behavior of rats living in mixed sex colonies, was an early pointer to the effect of environment on fear-associated behaviors. A major concern in terms of translational validity of most currently used tests is that females display lower levels of anxiety in “male” models, whereas anxiety-related psychopathology is far more common in women than men. However, in more ethologically relevant situations such as the risky closed economy, in an open field with cover, or when housed in a visible burrow system, females appear more anxious and risk averse than males (148, 183, 227, 244).

In women with anxiety disorders including panic and PTSD, anxiety, fear, and avoidance symptoms tend to increase during the premenstrual phase when progesterone is declining rapidly and estrogen is low (245, 246). In this respect the observation that responsiveness in tests of unconditioned fear behavior in rats mimics the clinical experience is pertinent, especially in the light of findings that the menstrual cycle in women influences principally emotion with limited effect on cognitive function (247). In female rats, unconditioned fear is significantly enhanced in diestrus 2 (33, 155, 179, 187) (similar to the premenstrual phase in women) whilst the cycle has inconsistent effects in tests employing conditioned threatening stimuli, which involve a learning component. The adverse symptoms experienced in the late luteal (premenstrual) phase may be considered an inappropriate over-reaction to everyday psychological stressors, which at other stages of their cycle do not trigger an adverse response. The clinical literature supports the hypothesis that premensrul dysphoric disorder pathophysiology is rooted in impaired GABA_A_-receptor response to dynamic fluctuations in allopregnanolone across the menstrual cycle, manifesting as affective symptoms and poor regulation of the physiologic stress response (245).

The importance of including females in all drug discovery protocols from the level of basic science using animal models to clinical trials in humans cannot be overstated. Greater standardization of experimental psychopharmacology protocols is required, in order to facilitate the search and characterization of novel anxiolytic agents for both sexes. Whilst females appear to respond in a qualitatively similar manner in most behavioral tests developed to model fear or anxiety in male rodents, it is becoming increasingly clear that male and female brains do not necessarily utilize the same neural mechanisms to achieve the same behavioral output. Moreover, behavioral responsiveness and drug action in females may be influenced by the changing chemical milieu of the brain during the estrous cycle. Drug development must be tailored to include female psychopharmacology with careful consideration of appropriate behavioral tests.

## Author Contributions

All authors listed have made a substantial, direct and intellectual contribution to the work, and approved it for publication.

## Conflict of Interest

The authors declare that the research was conducted in the absence of any commercial or financial relationships that could be construed as a potential conflict of interest.

## Publisher's Note

All claims expressed in this article are solely those of the authors and do not necessarily represent those of their affiliated organizations, or those of the publisher, the editors and the reviewers. Any product that may be evaluated in this article, or claim that may be made by its manufacturer, is not guaranteed or endorsed by the publisher.

## References

[1] Seeman MV. Psychopathology in women and men: focus on female hormones. Am J Psychiatry. (1997) 154:1641–7. 10.1176/ajp.154.12.1641 9396940

[2] Pigott TA. Gender differences in the epidemiology and treatment of anxiety disorders. J Clin Psychiatry. (1999) 60:4–15. 10487250

[3] Cover KK Maeng LY Lebrón-Milad K Milad MR. Mechanisms of estradiol in fear circuitry: implications for sex differences in psychopathology. Transl Psychiatry. (2014) 4:422. 10.1038/tp.2014.67 25093600 PMC4150242

[4] Ekhart C van Hunsel F Scholl J de Vries S van Puijenbroek E. Sex differences in reported adverse drug reactions of selective serotonin reuptake inhibitors. Drug Saf. (2018) 41:677–83. 10.1007/s40264-018-0646-2 29484612

[5] Reilly TJ Sagnay De La Bastida VC Joyce DW Cullen AE Mcguire P. Exacerbation of psychosis during the perimenstrual phase of the menstrual cycle: systematic review and meta-analysis. Schizophr Bull. (2020) 46:78–90. 10.1093/schbul/sbz030 31071226 PMC6942155

[6] Perna G Brambilla F Arancio C Bellodi L. Menstrual cycle-related sensitivity to 35% CO2 in panic patients. Biol Psychiatry. (1995) 37:528–32. 10.1016/0006-3223(94)00154-U 7619975

[7] Hughes RN. Sex still matters: has the prevalence of male-only studies of drug effects on rodent behaviour changed during the past decade? Behav Pharmacol. (2019) 30:95–9. 10.1097/FBP.0000000000000410 29847339

[8] McCarthy MM Woolley CS Arnold AP. Incorporating sex as a biological variable in neuroscience: what do we gain? Nat Rev Neurosci. (2017) 18:707–8. 10.1038/nrn.2017.137 29097784

[9] Inagaki H Mori Y. The emission of stress-induced 22-kHz calls in female rats is independent of testosterone levels. Horm Behav. (2015) 69:116–8. 10.1016/j.yhbeh.2015.01.001 25597917

[10] Giatti S Garcia-Segura LM Barreto GE Melcangi RC. Neuroactive steroids, neurosteroidogenesis and sex. Prog Neurobiol. (2019) 176:1–17. 10.1016/j.pneurobio.2018.06.007 29981391

[11] Li LH Wang ZC Yu J Zhang YQ. Ovariectomy results in variable changes in nociception, mood and depression in adult female rats. PLoS ONE. (2014) 9:e94312. 10.1371/journal.pone.0094312 24710472 PMC3978042

[12] Daendee S Thongsong B Kalandakanond-Thongsong S. Effects of time of estrogen deprivation on anxiety-like behavior and GABAA receptor plasticity in ovariectomized rats. Behav Brain Res. (2013) 246:86–93. 10.1016/j.bbr.2013.03.008 23499705

[13] Sarrel PM Sullivan SD Nelson LM. Hormone replacement therapy in young women with surgical primary ovarian insufficiency. Fertil Steril. (2016) 106:1580–7. 10.1016/j.fertnstert.2016.09.018 27793381 PMC5248494

[14] Brot MD Koob GF Britton KT. Anxiolytic effects of steroid hormones during the estrous cycle. interactions with ethanol. Recent Dev Alcohol. (1995) 12:243–59. 10.1007/0-306-47138-8_16 7624546

[15] Lebrón-Milad K Tsareva A Ahmed N Milad MR. Sex differences and estrous cycle in female rats interact with the effects of fluoxetine treatment on fear extinction. Behav Brain Res. (2013) 253:217–22. 10.1016/j.bbr.2013.07.024 23886596 PMC4106477

[16] Molina-Hernández M Téllez-Alcántara NP Olivera-López JI Jaramillo MT. Estrous cycle variation in anxiolytic-like effects of topiramate in Wistar rats in two animal models of anxiety-like behavior. Pharmacol Biochem Behav. (2013) 103:631–6. 10.1016/j.pbb.2012.11.002 23148913

[17] Rodríguez-Landa JF Vicente-Serna J Rodríguez-Blanco LA Rovirosa-Hernández MDJ García-Orduña F Carro-Juárez M. Montanoa frutescens and Montanoa grandiflora extracts reduce anxiety-like behavior during the metestrus-diestrus phase of the ovarian cycle in wistar rats. Biomed Res Int. (2014) 2014:938060. 10.1155/2014/938060 24800255 PMC3988800

[18] Rodríguez-Landa JF Guillén-Ruiz G Hernández-López F Cueto-Escobedo J Rivadeneyra-Domínguez E Bernal-Morales B. Chrysin reduces anxiety-like behavior through actions on GABAA receptors during metestrus-diestrus in the rat. Behav Brain Res. (2021) 397:112952. 10.1016/j.bbr.2020.112952 33017640

[19] De Paula Soares-Rachetti V De Sousa Pinto ÍA Santos RO André E Gavioli EC Lovick T. Short term, low dose fluoxetine blocks estrous cycle-linked changes in responsiveness to diazepam in female rats. J Psychopharmacol. (2016) 30:1062–8. 10.1177/0269881116636106 26956868

[20] Ennaceur A Chazot PL. Preclinical animal anxiety research – flaws and prejudices. Pharmacol Res Perspect. (2016) 4:1–37. 10.1002/prp2.223 27069634 PMC4804324

[21] Rodgers RJ. Animal models of “anxiety”: where next? Behav Pharmacol. (1997) 8:477–96. 10.1097/00008877-199711000-00003 9832964

[22] Stanford SC. Confusing preclinical (predictive) drug screens with animal “models” of psychiatric disorders, or “disorder-like” behaviour, is undermining confidence in behavioural neuroscience. J Psychopharmacol. (2017) 31:641–3. 10.1177/0269881116689260 28583048

[23] Chari T Griswold S Andrews NA Fagiolini M. The stage of the estrus cycle is critical for interpretation of female mouse social interaction behavior. Front Behav Neurosci. (2020) 14:113. 10.3389/fnbeh.2020.00113 32714163 PMC7340104

[24] Smith MS Freeman ME Neill JD. The control of progesterone secretion during the estrous cycle and early pseudopregnancy in the rat: prolactin, gonadotropin and steroid levels associated with rescue of the corpus luteum of pseudopregnancy. Endocrinology. (1975) 96:219–26. 10.1210/endo-96-1-219 1167352

[25] Brack KE Jeffery SMT Lovick TA. Cardiovascular and respiratory responses to a panicogenic agent in anaesthetised female Wistar rats at different stages of the oestrous cycle. Eur J Neurosci. (2006) 23:3309–18. 10.1111/j.1460-9568.2006.04881.x 16820020

[26] Meziane H Ouagazzal AM Aubert L Wietrzych M Krezel W. Estrous cycle effects on behavior of C57BL/6J and BALB/cByJ female mice: implications for phenotyping strategies. Genes Brain Behav. (2007) 6:192–200. 10.1111/j.1601-183X.2006.00249.x 16827921

[27] Pantier L Li J Christian C. Estrous cycle monitoring in mice with rapid data visualization and analysis. Bio-Protocol. (2019) 9:3354. 10.21769/BioProtoc.3354 32695847 PMC7372919

[28] Byers SL Wiles M V Dunn SL Taft RA. Mouse estrous cycle identification tool and images. PLoS ONE. (2012) 7:35538. 10.1371/journal.pone.0035538 22514749 PMC3325956

[29] Camacho-Arroyo I Piña-Medina AG Bello-Alvarez C Zamora-Sánchez CJ. Sex Hormones and Proteins Involved in Brain Plasticity. 1st ed. Elsevier Inc. (2020). 10.1016/bs.vh.2020.04.002 32723542

[30] Cora MC Kooistra L Travlos G. Vaginal cytology of the laboratory rat and mouse:review and criteria for the staging of the estrous cycle using stained vaginal smears. Toxicol Pathol. (2015) 43:776–93. 10.1177/0192623315570339 25739587 PMC11504324

[31] Mclean AC Valenzuela N Fai S Bennett SAL. Performing vaginal lavage, crystal violet staining, and vaginal cytological evaluation for mouse estrous cycle staging identification. J Vis Exp. (2012) e4389. 10.3791/4389 23007862 PMC3490233

[32] Butcher RL Collins WE Fugo NW. Plasma concentration of LH, FSH, prolactin, progesterone and estradiol-17beta throughout the 4-day estrous cycle of the rat. Endocrinology. (1974) 94:1704–8. 10.1210/endo-94-6-1704 4857496

[33] Devall AJ Santos JM Fry JP Honour JW Brandão ML Lovick TA. Elevation of brain allopregnanolone rather than 5-HT release by short term, low dose fluoxetine treatment prevents the estrous cycle-linked increase in stress sensitivity in female rats. Eur Neuropsychopharmacol. (2015) 25:113–23. 10.1016/j.euroneuro.2014.11.017 25498416

[34] Griffiths J Lovick T. Withdrawal from progesterone increases expression of α4, β1, and δ GABAA receptor subunits in neurons in the periaqueductal gray matter in female Wistar rats. J Comp Neurol. (2005) 486:89–97. 10.1002/cne.20540 15834956

[35] Smith SS Gong QH Li X Moran MH Bitran D Frye CA. Withdrawal from 3α-OH-5α-pregnan-20-one using a pseudopregnancy model alters the kinetics of hippocampal GABA(A)-gated current and increases the GABA(A) receptor α4 subunit in association with increased anxiety. J Neurosci. (1998) 18:5275–84. 10.1523/JNEUROSCI.18-14-05275.1998 9651210 PMC6793484

[36] Corpéchot C Collins BE Carey MP Tsouros A Robel P Fry JP. Brain neurosteroids during the mouse oestrous cycle. Brain Res. (1997) 766:276–80. 10.1016/S0006-8993(97)00749-X 9359616

[37] Bailey KJ. Diurnal progesterone rhythms in the female mouse. J Endocrinol. (1987) 112:15–21. 10.1677/joe.0.1120015 3819629

[38] Brown GP Courtney GA Marotta SF. A comparative study of adrenal progesterone secretion during the estrous cycles of hamsters and rats. Steroids. (1976) 28:283–94. 10.1016/0039-128X(76)90116-1 987624

[39] Carey MP Aniszewski CA F JP. Metabolism of progesterone in mouse brain. J Steroid Biochem Mol Biol. (1994) 50:213–7. 10.1016/0960-0760(94)90031-0 8049152

[40] Marcondes FK Miguel KJ Melo LL Spadari-Bratfisch RC. Estrous cycle influences the response of female rats in the elevated plus-maze test. Physiol Behav. (2001) 74:435–40. 10.1016/S0031-9384(01)00593-5 11790402

[41] Sugiyama M Yasunaga A Kobayashi R Fukasawa H Hashimoto O Kurusu S. Improvement in identification of pro-estrous mice by using a novel method of detecting vaginal mucous cells. Cell Tissue Res. (2021) 383:1183–90. 10.1007/s00441-020-03310-w 33242171

[42] Sano K Matsuda S Tohyama S Komura D Shimizu E Sutoh C. Deep learning-based classification of the mouse estrous cycle stages. Sci Rep. (2020) 10:11714. 10.1038/s41598-020-68611-0 32678183 PMC7366650

[43] Champlin AK Dorr DL Gates AH. Determining the stage of the estrous cycle in the mouse by the appearance of the vagina. Biol Reprod. (1973) 8:491–494. 10.1093/biolreprod/8.4.491 4736343

[44] Ratko M Habek N Kordić M DugandŽić A. The use of infrared technology as a novel approach for studies with female laboratory animals. Croat Med J. (2020) 61:346–53. 10.3325/cmj.2020.61.346 32881433 PMC7480751

[45] Ramos SD Lee JM Peuler JD. An inexpensive meter to measure differences in electrical resistance in the rat vagina during the ovarian cycle. J Appl Physiol. (2001) 91:667–70. 10.1152/jappl.2001.91.2.667 11457779

[46] Becegato M Meurer YSR Paiva-Santos MA Lima AC Marinho GF Bioni VS. Impaired discriminative avoidance and increased plasma corticosterone levels induced by vaginal lavage procedure in rats. Physiol Behav. (2021) 232:1–7. 10.1016/j.physbeh.2021.113343 33529686

[47] Barbaccia ML Roscetti G Trabucchi M Cuccheddu T Concas A Biggio G. Neurosteroids in the brain of handling-habituated and naive rats: effect of CO2 inhalation. Eur J Pharmacol. (1994) 261:317–20. 10.1016/0014-2999(94)90123-6 7813554

[48] Graeff FG Zangrossi H. Animal models of anxiety disorders. In: D'Haenen H den Boer JA Willner P, editors. Biological Psychiatry (John Wiley & Sons Ltd.). p. 877–93. 10.1002/0470854871.chxix1

[49] McNaughton N Corr PJ. A two-dimensional neuropsychology of defense: fear/anxiety and defensive distance. Neurosci Biobehav Rev. (2004) 28:285–305. 10.1016/j.neubiorev.2004.03.005 15225972

[50] Fendt M Parsons MH Apfelbach R Carthey AJR Dickman CR Endres T. Context and trade-offs characterize real-world threat detection systems: a review and comprehensive framework to improve research practice and resolve the translational crisis. Neurosci Biobehav Rev. (2020) 115:25–33. 10.1016/j.neubiorev.2020.05.002 32439371

[51] Blanchard DC Summers CH Blanchard RJ. The role of behavior in translational models for psychopathology: functionality and dysfunctional behaviors. Neurosci Biobehav Rev. (2013) 37:1567–77. 10.1016/j.neubiorev.2013.06.008 23791787 PMC3800172

[52] Bourin M. Animal models for screening anxiolytic-like drugs: a perspective. Dialogues Clin Neurosci. (2015) 17:295–303. 10.31887/DCNS.2015.17.3/mbourin 26487810 PMC4610614

[53] Campos AC Fogaça M V Aguiar DC Guimarães FS. Animal models of anxiety disorders and stress. Rev Bras Psiquiatr. (2013) 35 (Suppl. 2):S101–11. 10.1590/1516-4446-2013-1139 24271222

[54] Handley SL Mithani S. Effects of alpha-adrenoceptor agonists and antagonists in a maze-exploration model of 'fear'-motivated behaviour. Naunyn Schmiedebergs Arch Pharmacol. (1984) 327:1–5. 10.1007/BF00504983 6149466

[55] Pellow S Chopin P File SE Briley M. Validation of open : closed arm entries in an elevated plus-maze as a measure of anxiety in the rat. J Neurosci Methods. (1985) 14:149–67. 10.1016/0165-0270(85)90031-7 2864480

[56] Himanshu Dharmila Sarkar D Nutan. A review of behavioral tests to evaluate different types of anxiety and anti-anxiety effects. Clin Psychopharmacol Neurosci. (2020) 18:341–51. 10.9758/cpn.2020.18.3.341 32702213 PMC7382999

[57] La-Vu M Tobias BC Schuette PJ Adhikari A. To approach or avoid: an introductory overview of the study of anxiety using rodent assays. Front Behav Neurosci. (2020) 14:145. 10.3389/fnbeh.2020.00145 33005134 PMC7479238

[58] Gogokhia N Japaridze N Tizabi Y Pataraya L Zhvania MG. Gender differences in anxiety response to high intensity white noise in rats. Neurosci Lett. (2021) 742:135543. 10.1016/j.neulet.2020.135543 33278506

[59] Johnston AL File SE. Sex differences in animal tests of anxiety. Physiol Behav. (1991) 49:245–50. 10.1016/0031-9384(91)90039-Q 2062894

[60] Knight P Chellian R Wilson R Behnood-Rod A Panunzio S Bruijnzeel AW. Sex differences in the elevated plus-maze test and large open field test in adult Wistar rats. Pharmacol Biochem Behav. (2021) 204:173168. 10.1016/j.pbb.2021.173168 33684454 PMC8130853

[61] Scholl JL Afzal A Fox LC Watt MJ Forster GL. Sex differences in anxiety-like behaviors in rats. Physiol Behav. (2019) 211:112670. 10.1016/j.physbeh.2019.112670 31487491

[62] Frick KM Burlingame LA Arters JA Berger-Sweeney J. Reference memory, anxiety and estrous cyclicity in C57BL/6NIA mice are affected by age and sex. Neuroscience. (2000) 95:293–307. 10.1016/S0306-4522(99)00418-2 10619486

[63] Tucker LB McCabe JT. Behavior of male and female C57Bl/6J mice is more consistent with repeated trials in the elevated zero maze than in the elevated plus maze. Front Behav Neurosci. (2017) 11:13. 10.3389/fnbeh.2017.00013 28184191 PMC5266707

[64] D'Souza D Sadananda M. Estrous cycle phase-dependent changes in anxiety-and depression-like profiles in the late adolescent Wistar-Kyoto rat. Ann Neurosci. (2017) 24:136–45. 10.1159/000477151 28867895 PMC5566678

[65] Díaz-Véliz G Dussaubat N Mora S. Ketanserin effects on rat behavioral responses: modifications by the estrous cycle, ovariectomy and estradiol replacement. Pharmacol Biochem Behav. (1997) 57:687–92. 10.1016/S0091-3057(96)00394-2 9258995

[66] Frye CA Petralia SM Rhodes ME. Estrous cycle and sex differences in performance on anxiety tasks coincide with increases in hippocampal progesterone and 3α,5α-THP. Pharmacol Biochem Behav. (2000) 67:587–96. 10.1016/S0091-3057(00)00392-0 11164090

[67] Frye CA Wawrzycki JA. Effect of prenatal stress and gonadal hormone condition on depressive behaviors of female and male rats. Horm Behav. (2003) 44:319–26. 10.1016/S0018-506X(03)00159-4 14613726

[68] Koonce CJ Walf AA Frye CA. Type 1 5α-reductase may be required for estrous cycle changes in affective behaviors of female mice. Behav Brain Res. (2012) 226:376–80. 10.1016/j.bbr.2011.09.028 21946309 PMC3381506

[69] Mora S Dussaubat N Díaz-Véliz G. Effects of the estrous cycle and ovarian hormones on behavioral indices of anxiety in female rats. Psychoneuroendocrinology. (1996) 21:609–20. 10.1016/S0306-4530(96)00015-7 9044444

[70] Walf AA Frye CA. Estradiol decreases anxiety behavior and enhances inhibitory avoidance and gestational stress produces opposite effects. Stress. (2007) 10:251–60. 10.1080/00958970701220416 17613939

[71] Fernández-Guasti A Martínez-Mota L Estrada-Camarena E Contreras CM López-Rubalcava C. Chronic treatment with desipramine induces an estrous cycle-dependent anxiolytic-like action in the burying behavior, but not in the elevated plus-maze test. Pharmacol Biochem Behav. (1999) 63:13–20. 10.1016/S0091-3057(98)00231-7 10340518

[72] Nin MS Couto-Pereira NS Souza MF Azeredo LA Ferri MK Dalprá WL. Anxiolytic effect of clonazepam in female rats: grooming microstructure and elevated plus maze tests. Eur J Pharmacol. (2012) 684:95–101. 10.1016/j.ejphar.2012.03.038 22487059

[73] Sakae DY Sakae TM Paschoalini MA Faria MS. Relative luminosity in the plus maze upon the exploratory behaviour of female Wistar rats. Arq Neuropsiquiatr. (2015) 73:601–6. 10.1590/0004-282X20150088 26200055

[74] Datta S Samanta D Sinha P Chakrabarti N. Gender features and estrous cycle variations of nocturnal behavior of mice after a single exposure to light at night. Physiol Behav. (2016) 164:113–22. 10.1016/j.physbeh.2016.05.049 27241632

[75] Walf AA Koonce C Manley K Frye CA. Proestrous compared to diestrous wildtype, but not estrogen receptor beta knockout, mice have better performance in the spontaneous alternation and object recognition tasks and reduced anxiety-like behavior in the elevated plus and mirror maze. Behav Brain Res. (2009) 196:254–60. 10.1016/j.bbr.2008.09.016 18926853 PMC2614898

[76] Gangitano D Salas R Teng Y Perez E De Biasi M. Progesterone modulation of α5 nAChR subunits influences anxiety-related behavior during estrus cycle. Genes Brain Behav. (2009) 8:398–406. 10.1111/j.1601-183X.2009.00476.x 19220484 PMC2712346

[77] Carobrez AP Bertoglio LJ. Ethological and temporal analyses of anxiety-like behavior: the elevated plus-maze model 20 years on. Neurosci Biobehav Rev. (2005) 29:1193–205. 10.1016/j.neubiorev.2005.04.017 16084592

[78] Pereira LO Da Cunha IC Neto JM Paschoalini MA Faria MS. The gradient of luminosity between open/enclosed arms, and not the absolute level of Lux, predicts the behaviour of rats in the plus maze. Behav Brain Res. (2005) 159:55–61. 10.1016/j.bbr.2004.10.002 15794998

[79] Albani SH Andrawis MM Abella RJH Fulghum JT Vafamand N Dumas TC. Behavior in the elevated plus maze is differentially affected by testing conditions in rats under and over three weeks of age. Front Behav Neurosci. (2015) 9:31. 10.3389/fnbeh.2015.00031 25741257 PMC4330883

[80] Andrade MMM Tomé MF Santiago ES Lúcia-Santos A De Andrade TGCS. Longitudinal study of daily variation of rats' behavior in the elevated plus-maze. Physiol Behav. (2003) 78:125–33. 10.1016/S0031-9384(02)00941-1 12536019

[81] Bertoglio LJ Carobrez AP. Behavioral profile of rats submitted to session 1-session 2 in the elevated plus-maze during diurnal/nocturnal phases and under different illumination conditions. Behav Brain Res. (2002) 132:135–43. 10.1016/S0166-4328(01)00396-5 11997144

[82] Bilu C Kronfeld-Schor N. Effects of circadian phase and melatonin injection on anxiety-like behavior in nocturnal and diurnal rodents. Chronobiol Int. (2013) 30:828–36. 10.3109/07420528.2013.773439 23750894

[83] Griebel G Moreau JL Jenck F Misslin R Martin JR. Acute and chronic treatment with 5-HT reuptake inhibitors differentially modulate emotional responses in anxiety models in rodents. Psychopharmacology. (1994) 113:463–70. 10.1007/BF02245224 7862860

[84] Haller J Alicki M. Current animal models of anxiety, anxiety disorders, and anxiolytic drugs. Curr Opin Psychiatry. (2012) 25:59–64. 10.1097/YCO.0b013e32834de34f 22156938

[85] Jones N King SM. Influence of circadian phase and test illumination on pre-clinical models of anxiety. Physiol Behav. (2001) 72:99–106. 10.1016/S0031-9384(00)00388-7 11239986

[86] Verma P Hellemans KGC Choi FY Yu W Weinberg J. Circadian phase and sex effects on depressive/anxiety-like behaviors and HPA axis responses to acute stress. Physiol Behav. (2010) 99:276–85. 10.1016/j.physbeh.2009.11.002 19932127 PMC2856664

[87] Handley SL McBlane JW. An assessment of the elevated X-maze for studying anxiety and anxiety-modulating drugs. J Pharmacol Toxicol Methods. (1993) 29:129–38. 10.1016/1056-8719(93)90063-K 8103377

[88] Pinheiro SH Zangrossi-jr H Del-ben CM. Elevated mazes as animal models of anxiety : effects of serotonergic agents. An Acad Bras Cienc. (2007) 79:71–85. 10.1590/S0001-37652007000100010 17401477

[89] Zangrossi H Graeff FG. Behavioral validation of the elevated T-maze, a new animal model of anxiety. Brain Res Bull. (1997) 44:1–5. 10.1016/S0361-9230(96)00381-4 9288825

[90] Graeff FG Viana MB Tomaz C. The elevated T maze, a new experimental model of anxiety and memory: effect of diazepam. Brazilian J Med Biol Res. (1993) 26:67–70. 8220269

[91] Viana MB Tomaz C Graeff FG. The elevated T-maze: a new animal model of anxiety and memory. Pharmacol Biochem Behav. (1994) 49:549–54. 10.1016/0091-3057(94)90067-1 7862706

[92] Zangrossi H Graeff FG. Serotonin in anxiety and panic: contributions of the elevated T-maze. Neurosci Biobehav Rev. (2014) 46:397–406. 10.1016/j.neubiorev.2014.03.007 24657635

[93] Gouveia A Dos Santos UD Felisbino FE De Afonseca TL Antunes G Morato S. Influence of the estrous cycle on the behavior of rats in the elevated T-maze. Behav Processes. (2004) 67:167–71. 10.1016/j.beproc.2004.03.018 15240054

[94] Rocinholi LF Landeira-Fernandez J. Anxiety-like behavior in weanling and young adult male and female malnourished rats. Physiol Behav. (2011) 102:13–6. 10.1016/j.physbeh.2010.09.017 20888355

[95] Santos RO de Assunção GLM de Medeiros DMB de Sousa Pinto ÍA de Barros KS Soares BL. Evaluation of the effect of acute sibutramine in female rats in the elevated T-Maze and elevated plus-maze tests. Basic Clin Pharmacol Toxicol. (2014) 114:181–7. 10.1111/bcpt.12131 24034271

[96] Slotten HA Kalinichev M Hagan JJ Marsden CA Fone KCF. Long-lasting changes in behavioural and neuroendocrine indices in the rat following neonatal maternal separation: gender-dependent effects. Brain Res. (2006) 1097:123–32. 10.1016/j.brainres.2006.04.066 16730678

[97] O'Leary TP Gunn RK Brown RE. What are we measuring when we test strain differences in anxiety in mice? Behav Genet. (2013) 43:34–50. 10.1007/s10519-012-9572-8 23288504

[98] Ramos A. Animal models of anxiety: do I need multiple tests? Trends Pharmacol Sci. (2008) 29:493–8. 10.1016/j.tips.2008.07.005 18755516

[99] Ramos A Pereira E Martins GC Wehrmeister TD Izídio GS. Integrating the open field, elevated plus maze and light/dark box to assess different types of emotional behaviors in one single trial. Behav Brain Res. (2008) 193:277–88. 10.1016/j.bbr.2008.06.007 18590774

[100] Archer J. Rodent sex differences in emotional and related behavior. Behav Biol. (1975) 14:451–79. 10.1016/S0091-6773(75)90636-7 1100044

[101] Beatty WW Fessler RG. Ontogeny of sex differences in open-field behavior and sensitivity to electric shock in the rat. Physiol Behav. (1976) 16:413–7. 10.1016/0031-9384(76)90319-X 959343

[102] Blizard DA. Sex differences in running-wheel behaviour in the rat: the inductive and activational effects of gonadal hormones. Anim Behav. (1975) 14:601–8. 10.1016/0031-9384(75)90188-2 1135316

[103] Masur J Schutz MT Boerngen R. Gender differences in open-field behavior as a function of age. Dev Psychobiol. (1980) 13:107–10. 10.1002/dev.420130202 7358217

[104] Fritz AK Amrein I Wolfer DP. Similar reliability and equivalent performance of female and male mice in the open field and water-maze place navigation task. Am J Med Genet Part C Semin Med Genet. (2017) 175:380–91. 10.1002/ajmg.c.31565 28654717 PMC5638061

[105] Milner LC Crabbe JC. Three murine anxiety models: results from multiple inbred strain comparisons. Genes Brain Behav. (2008) 7:496–505. 10.1111/j.1601-183X.2007.00385.x 18182070

[106] Hiroi R Neumaier JF. Differential effects of ovarian steroids on anxiety versus fear as measured by open field test and fear-potentiated startle. Behav Brain Res. (2006) 166:93–100. 10.1016/j.bbr.2005.07.021 16154649

[107] Zhao Y Bijlsma EY Verdouw MP Groenink L. No effect of sex and estrous cycle on the fear potentiated startle response in rats. Behav Brain Res. (2018) 351:24–33. 10.1016/j.bbr.2018.05.022 29803653

[108] Miller CK Halbing AA Patisaul HB Meitzen J. Interactions of the estrous cycle, novelty, and light on female and male rat open field locomotor and anxiety-related behaviors. Physiol Behav. (2021) 228:113203. 10.1016/j.physbeh.2020.113203 33045240 PMC7736204

[109] Dichter GS Brunelli SA Hofer MA. Elevated plus-maze behavior in adult offspring of selectively bred rats. Physiol Behav. (1996) 60:299–304. 10.1016/0031-9384(95)02222-8 8804680

[110] Elliott BM Faraday MM Phillips JM Grunberg NE. Effects of nicotine on elevated plus maze and locomotor activity in male and female adolescent and adult rats. Pharmacol Biochem Behav. (2004) 77:21–8. 10.1016/j.pbb.2003.09.016 14724038

[111] Gray JA Lalljee B. Sex differences in emotional behaviour in the rat: correlation between open-field defecation and active avoidance. Anim Behav. (1974) 22:856–61. 10.1016/0003-3472(74)90008-6 4477944

[112] Harrington GM. Strain differences in open-field behavior of the rat. Psychon Sci. (1972) 27:51–3. 10.3758/BF033288879829298

[113] Steenbergen HL Farabollini F Heinsbroek RPW. Sex-dependent effects of aversive stimulation on holeboard and elevated plus-maze behavior. Behav Brain Res. (1991) 43:159–65. 10.1016/S0166-4328(05)80066-X 1867757

[114] Kastenberger I Schwarzer C. GPER1 (GPR30) knockout mice display reduced anxiety and altered stress response in a sex and paradigm dependent manner. Horm Behav. (2014) 66:628–36. 10.1016/j.yhbeh.2014.09.001 25236887 PMC4213071

[115] Crawley J Goodwin FK. Preliminary report of a simple animal behavior model for the anxiolytic effects of benzodiazepines. Pharmacol Biochem Behav. (1980) 13:167–70. 10.1016/0091-3057(80)90067-2 6106204

[116] Crawley JN. Exploratory behavior models of anxiety in mice. Neurosci Biobehav Rev. (1985) 9:37–44. 10.1016/0149-7634(85)90030-2 2858080

[117] Amodeo LR Wills DN Sanchez-Alavez M Nguyen W Conti B Ehlers CL. Intermittent voluntary ethanol consumption combined with ethanol vapor exposure during adolescence increases drinking and alters other behaviors in adulthood in female and male rats. Alcohol. (2018) 73:57–66. 10.1016/j.alcohol.2018.04.003 30293056 PMC6193864

[118] Domonkos E Borbélyová V Csongová M Bosý M Ka M Ostatníková D. Hormones and Behavior Sex differences and sex hormones in anxiety-like behavior of aging rats. Horm Behav. (2017) 93:159–65. 10.1016/j.yhbeh.2017.05.019 28576648

[119] Ferguson SA Berry KJ. Chronic oral treatment with isotretinoin alters measures of activity but not anxiety in male and female rats. Neurotoxicol Teratol. (2010) 32:573–8. 10.1016/j.ntt.2010.03.009 20381607

[120] Hughes RN Hamilton JJ. Sex-dependent modification by chronic caffeine of acute methamphetamine effects on anxiety-related behavior in rats. Behav Brain Res. (2018) 345:30–8. 10.1016/j.bbr.2018.02.013 29476897

[121] Lovelock DF Deak T. Acute stress imposed during adolescence yields heightened anxiety in Sprague Dawley rats that persists into adulthood: sex differences and potential involvement of the Medial Amygdala. Brain Res. (2019) 1723:146392. 10.1016/j.brainres.2019.146392 31446016 PMC6766421

[122] Slattery DA Neumann ID. Chronic icv oxytocin attenuates the pathological high anxiety state of selectively bred Wistar rats. Neuropharmacology. (2010) 58:56–61. 10.1016/j.neuropharm.2009.06.038 19589349

[123] Tsuda MC Mahdi S Namchuk A Wu TJ Lucki I. Vendor differences in anxiety-like behaviors in female and male Sprague Dawley rats. Physiol Behav. (2020) 227:113131. 10.1016/j.physbeh.2020.113131 32791181

[124] Inglis A Shibin S Ubungen R Farooq S Mata P Thiam J. Strain and sex-based glucocentric & behavioral differences between KK/HlJ and C57BL/6J mice. Physiol Behav. (2019) 210:112646. 10.1016/j.physbeh.2019.112646 31400379

[125] Majidi-Zolbanin J Azarfarin M Samadi H Enayati M Salari AA. Adolescent fluoxetine treatment decreases the effects of neonatal immune activation on anxiety-like behavior in mice. Behav Brain Res. (2013) 250:123–32. 10.1016/j.bbr.2013.05.003 23669137

[126] Makinson R Lloyd K Rayasam A McKee S Brown A Barila G. Intrauterine inflammation induces sex-specific effects on neuroinflammation, white matter, and behavior. Brain Behav Immun. (2017) 66:277–88. 10.1016/j.bbi.2017.07.016 28739513 PMC6916731

[127] Meseguer Henarejos AB Popović N Bokonjić D Morales-Delgado N Alonso A Caballero Bleda M. Sex and time-of-day impact on anxiety and passive avoidance memory strategies in mice. Front Behav Neurosci. (2020) 14:68. 10.3389/fnbeh.2020.00068 32523516 PMC7261894

[128] Rilett KC Friedel M Ellegood J MacKenzie RN Lerch JP Foster JA. Loss of T cells influences sex differences in behavior and brain structure. Brain Behav Immun. (2015) 46:249–60. 10.1016/j.bbi.2015.02.016 25725160

[129] Wiersielis KR Adams S Yasrebi A Conde K Roepke TA. Maternal exposure to organophosphate flame retardants alters locomotor and anxiety-like behavior in male and female adult offspring. Horm Behav. (2020) 122:104759. 10.1016/j.yhbeh.2020.104759 32320692 PMC8530209

[130] Yohn CN Ashamalla SA Bokka L Gergues MM Garino A Samuels BA. Social instability is an effective chronic stress paradigm for both male and female mice. Neuropharmacology. (2019) 160:107780. 10.1016/j.neuropharm.2019.107780 31536736 PMC6935299

[131] Åhlgren J Voikar V. Housing mice in the individually ventilated or open cages—Does it matter for behavioral phenotype? Genes, Brain Behav. (2019) 18:1–2. 10.1111/gbb.12564 30848040 PMC6849734

[132] Võikar V Kõks S Vasar E Rauvala H. Strain and gender differences in the behavior of mouse lines commonly used in transgenic studies. Physiol Behav. (2001) 72:271–81. 10.1016/S0031-9384(00)00405-4 11240006

[133] Bishnoi IR Ossenkopp KP Kavaliers M. Sex and age differences in locomotor and anxiety-like behaviors in rats: from adolescence to adulthood. Dev Psychobiol. (2021) 63:496–511. 10.1002/dev.22037 33047845

[134] Fleming W Jones Q Chandra U Saini A Walker D Francis R. Withdrawal from brief repeated alcohol treatment in adolescent and adult male and female rats. Alcohol Clin Exp Res. (2019) 43:204–11. 10.1111/acer.13936 30566247 PMC6370488

[135] Carreira MB Cossio R Britton GB. Individual and sex differences in high and low responder phenotypes. Behav Processes. (2017) 136:20–7. 10.1016/j.beproc.2017.01.006 28088551

[136] Guo M Wu CF Liu W Yang JY Chen D. Sex difference in psychological behavior changes induced by long-term social isolation in mice. Prog Neuro-Psychopharmacology Biol Psychiatry. (2004) 28:115–21. 10.1016/j.pnpbp.2003.09.027 14687865

[137] Luckhart C Philippe TJ Le François B Vahid-Ansari F Geddes SD Béïque JC. Sex-dependent adaptive changes in serotonin-1A autoreceptor function and anxiety in Deaf1-deficient mice. Mol Brain. (2016) 9:1–2. 10.1186/s13041-016-0254-y 27488351 PMC4973060

[138] Turgeon SM Kim D Pritchard M Salgado S Thaler A. The effects of phencyclidine (PCP) on anxiety-like behavior in the elevated plus maze and the light-dark exploration test are age dependent, sexually dimorphic, and task dependent. Pharmacol Biochem Behav. (2011) 100:191–8. 10.1016/j.pbb.2011.08.017 21889525

[139] Budylin T Guariglia SR Duran LI Behring BM Shaikh Z Neuwirth LS. Ultrasonic vocalization sex differences in 5-HT1A-R deficient mouse pups: predictive phenotypes associated with later-life anxiety-like behaviors. Behav Brain Res. (2019) 373:112062. 10.1016/j.bbr.2019.112062 31288061

[140] Gillette R Reilly MP Topper VY Thompson LM Crews D Gore AC. Anxiety-like behaviors in adulthood are altered in male but not female rats exposed to low dosages of polychlorinated biphenyls in utero. Horm Behav. (2017) 87:8–15. 10.1016/j.yhbeh.2016.10.011 27794483 PMC5603326

[141] Hughes RN Hancock NJ. Strain-dependent effects of acute caffeine on anxiety-related behavior in PVG/c, Long-Evans and Wistar rats. Pharmacol Biochem Behav. (2016) 140:51–61. 10.1016/j.pbb.2015.11.005 26577750

[142] Leonardo Jimenez Chavez C Coelho MA Brewin LW Swauncy I Tran T Albanese T. Incubation of negative affect during protracted alcohol withdrawal is age-, but not sex-selective. Brain Sci. (2020) 10:1–27. 10.3390/brainsci10060405 32604806 PMC7348966

[143] Ramos A Kangerski AL Basso PF Da Silva Santos JE Assreuy J Vendruscolo LF. Evaluation of Lewis and SHR rat strains as a genetic model for the study of anxiety and pain. Behav Brain Res. (2002) 129:113–23. 10.1016/S0166-4328(01)00337-0 11809502

[144] Grundwald NJ Brunton PJ. Prenatal stress programs neuroendocrine stress responses and affective behaviors in second generation rats in a sex-dependent manner. Psychoneuroendocrinology. (2015) 62:204–16. 10.1016/j.psyneuen.2015.08.010 26318631 PMC4642655

[145] Zuluaga MJ Agrati D Pereira M Uriarte N Fernández-Guasti A Ferreira A. Experimental anxiety in the black and white model in cycling, pregnant and lactating rats. Physiol Behav. (2005) 84:279–86. 10.1016/j.physbeh.2004.12.004 15708779

[146] Kokras N Dalla C Sideris AC Dendi A Mikail HG Antoniou K. Behavioral sexual dimorphism in models of anxiety and depression due to changes in HPA axis activity. Neuropharmacology. (2012) 62:436–45. 10.1016/j.neuropharm.2011.08.025 21884710

[147] Simola N Granon S. Ultrasonic vocalizations as a tool in studying emotional states in rodent models of social behavior and brain disease. Neuropharmacology. (2019) 159:107420. 10.1016/j.neuropharm.2018.11.008 30445100

[148] Blanchard DC Shepherd JK Carobrez ADP Blanchard RJ. Sex effects in defensive behavior: baseline differences and drug interactions. Neurosci Biobehav Rev. (1991) 15:461–8. 10.1016/S0149-7634(05)80132-0 1686485

[149] Blanchard RJ Yudko EB Rodgers RJ Blanchard DC. Defense system psychopharmacology: an ethological approach to the pharmacology of fear and anxiety. Behav Brain Res. (1993) 58:155–65. 10.1016/0166-4328(93)90100-5 7907880

[150] Blanchard DC Griebel G Blanchard RJ. Mouse defensive behaviors: pharmacological and behavioral assays for anxiety and panic. Neurosci Biobehav Rev. (2001) 25:205–18. 10.1016/S0149-7634(01)00009-4 11378177

[151] Litvin Y Blanchard DC Blanchard RJ. Rat 22 kHz ultrasonic vocalizations as alarm cries. Behav Brain Res. (2007) 182:166–72. 10.1016/j.bbr.2006.11.038 17173984

[152] Portfors C V. Types and functions of ultrasonic vocalizations in laboratory rats and mice. J Am Assoc Lab Anim Sci. (2007) 46:28–34. 17203913

[153] Machado Figueiredo R de Carvalho MC Brandão ML Lovick TA. Short-term, low-dose fluoxetine prevents oestrous cycle-linked increase in anxiety-like behaviour in female rats. J Psychopharmacol. (2019) 33:548–57. 10.1177/0269881119841833 31012390

[154] Browning JR Whiteman AC Leung LY Lu XCM Shear DA. Air-puff induced vocalizations: a novel approach to detecting negative affective state following concussion in rats. J Neurosci Methods. (2017) 275:45–9. 10.1016/j.jneumeth.2016.10.017 27984100

[155] Inagaki H Sato J. Air puff-induced 22-kHz calls in F344 rats. Physiol Behav. (2016) 155:237–41. 10.1016/j.physbeh.2015.12.022 26723270

[156] Drugan RC Warner TA Papallo TA Castracane LL Stafford NP. Ultrasonic vocalizations during intermittent swim stress forecasts resilience in subsequent forced swim and spatial learning tests. Behav Brain Res. (2014) 259:41–4. 10.1016/j.bbr.2013.10.029 24475493

[157] Zambetti PR Schuessler BP Kim JJ. Sex differences in foraging rats to naturalistic aerial predator stimuli. iScience. (2019) 16:442–52. 10.1016/j.isci.2019.06.011 31229893 PMC6593150

[158] Taylor JO Urbano CM Cooper BG. Differential patterns of constant frequency 50 and 22 khz usv production are related to intensity of negative affective state. Behav Neurosci. (2017) 131:115–26. 10.1037/bne0000184 28054809

[159] Wöhr M Schwarting RKW. Affective communication in rodents: ultrasonic vocalizations as a tool for research on emotion and motivation. Cell Tissue Res. (2013) 354:81–97. 10.1007/s00441-013-1607-9 23576070

[160] Beck JG Ohtake PJ Shipherd JC. Exaggerated anxiety is not unique to CO2 in panic disorder: a comparison of hypercapnic and hypoxic challenges. J Abnorm Psychol. (1999) 108:473–82. 10.1037/0021-843X.108.3.473 10466271

[161] Leibold NK Van Den Hove DLA Viechtbauer W Buchanan GF Goossens L Lange I. CO2 exposure as translational cross-species experimental model for panic. Transl Psychiatry. (2016) 6:e885–9. 10.1038/tp.2016.162 27598969 PMC5048202

[162] Perna G Casolari A Bussi R Cucchi M Arancio C Bellodi L. Comparison of 35% carbon dioxide reactivity between panic disorder and eating disorder. Psychiatry Res. (2004) 125:277–83. 10.1016/j.psychres.2003.12.017 15051188

[163] Johnson PL Federici LM Shekhar A. Etiology, triggers and neurochemical circuits associated with unexpected, expected, and laboratory-induced panic attacks. Neurosci Biobehav Rev. (2014) 46:429–54. 10.1016/j.neubiorev.2014.07.027 25130976 PMC4252820

[164] Kinkead R Tenorio L Drolet G Bretzner F Gargaglioni L. Respiratory manifestations of panic disorder in animals and humans: a unique opportunity to understand how supramedullary structures regulate breathing. Respir Physiol Neurobiol. (2014) 204:3–13. 10.1016/j.resp.2014.06.013 25038523

[165] Borkowski AH Barnes DC Blanchette DR Castellanos FX Klein DF Wilson DA. Interaction between delta opioid receptors and benzodiazepines in CO 2-induced respiratory responses in mice. Brain Res. (2011) 1396:54–9. 10.1016/j.brainres.2011.04.042 21561601 PMC3104108

[166] Cittaro D Lampis V Luchetti A Coccurello R Guffanti A Felsani A. Histone modifications in a mouse model of early adversities and panic disorder: role for Asic1 and neurodevelopmental genes. Sci Rep. (2016) 6:25131. 10.1038/srep25131 27121911 PMC4848503

[167] Dumont FS Biancardi V Kinkead R. Hypercapnic ventilatory response of anesthetized female rats subjected to neonatal maternal separation: insight into the origins of panic attacks? Respir Physiol Neurobiol. (2011) 175:288–95. 10.1016/j.resp.2010.12.004 21147276

[168] Kinkead R Montandon G Bairam A Lajeunesse Y Horner R. Neonatal maternal separation disrupts regulation of sleep and breathing in adult male rats. Sleep. (2009) 32:1611–20. 10.1093/sleep/32.12.1611 20041597 PMC2786045

[169] Luchetti A Oddi D Lampis V Centofante E Felsani A Battaglia M. Early handling and repeated cross-fostering have opposite effect on mouse emotionality. Front Behav Neurosci. (2015) 9:93. 10.3389/fnbeh.2015.00093 25954170 PMC4404916

[170] Bonaventure P Dugovic C Shireman B Preville C Yun S Lord B. Evaluation of JNJ-54717793 a novel brain penetrant selective orexin 1 receptor antagonist in two rat models of panic attack provocation. Front Pharmacol. (2017) 8:357. 10.3389/fphar.2017.00357 28649201 PMC5465257

[171] Cuccheddu T Floris S Serra M Luisa Porceddu M Sanna E Biggio G. Proconflict effect of carbon dioxide inhalation in rats. Life Sci. (1995) 56:321–4. 10.1016/0024-3205(95)00093-3 8614250

[172] Duszczyk M Gamdzyk M Ziembowicz A Boguszewski PM Łazarewicz JW Salińska E. Antidepressant-like and anxiolytic-like effects of mild hypobaric hypoxia in mice: possible involvement of neuropeptide Y. Acta Neurobiol Exp. (2015) 75:364–71. 26994415

[173] Hickman DL Fitz SD Bernabe CS Caliman IF Haulcomb MM Federici LM. Evaluation of low versus high volume per minute displacement CO2 methods of euthanasia in the induction and duration of panic-associated behavior and physiology. Animals. (2016) 6:45. 10.3390/ani6080045 27490573 PMC4997270

[174] Kiray M Sisman AR Camsari UM Evren M Dayi A Baykara B. Effects of carbon dioxide exposure on early brain development in rats. Biotech Histochem. (2014) 89:371–83. 10.3109/10520295.2013.872298 24476563

[175] Gargaglioni LH Marques DA Patrone LGA. Sex differences in breathing. Comp Biochem Physiol -Part A Mol Integr Physiol. (2019) 238:110543. 10.1016/j.cbpa.2019.110543 31445081

[176] Spiacci A de Oliveira Sergio T da Silva GSF Glass ML Schenberg LC Garcia-Cairasco N. Serotonin in the dorsal periaqueductal gray inhibits panic-like defensive behaviors in rats exposed to acute hypoxia. Neuroscience. (2015) 307:191–8. 10.1016/j.neuroscience.2015.08.045 26319117

[177] Spiacci A Vilela-Costa HH Sant'Ana AB Fernandes GG Frias AT da Silva GSF. Panic-like escape response elicited in mice by exposure to CO2, but not hypoxia. Prog Neuro-Psychopharmacology Biol Psychiatry. (2018) 81:178–86. 10.1016/j.pnpbp.2017.10.018 29111406

[178] Batistela MF Vilela-Costa HH Frias AT Lovick TA Zangrossi H Jr. Enhanced responsiveness to hypoxic panicogenic challenge in female rats in late diestrus is suppressed by short term, low dose fluoxetine: involvement of the dorsal raphe nucleus and the dorsal periaqueductal grey. J Psychopharmacol. (2021). 10.1177/02698811211058986 34872406

[179] Nillni YI Rohan KJ Zvolensky MJ. The role of menstrual cycle phase and anxiety sensitivity in catastrophic misinterpretation of physical symptoms during a CO2 challenge. Arch Womens Ment Health. (2012) 15:413–22. 10.1007/s00737-012-0302-2 22923028 PMC3495998

[180] Sigmon ST Dorhofer DM Rohan KJ Hotovy LA Boulard NE Fink CM. Psychophysiological, somatic, and affective changes across the menstrual cycle in women with panic disorder. J Consult Clin Psychol. (2000) 68:425–31. 10.1037/0022-006X.68.3.425 10883559

[181] Blanchard DC Griebel G Pobbe R Blanchard RJ. Risk assessment as an evolved threat detection and analysis process. Neurosci Biobehav Rev. (2011) 35:991–8. 10.1016/j.neubiorev.2010.10.016 21056591

[182] Hubbard DT Blanchard DC Yang M Markham CM Gervacio A Chun-I L. Development of defensive behavior and conditioning to cat odor in the rat. Physiol Behav. (2004) 80:525–30. 10.1016/j.physbeh.2003.10.006 14741237

[183] Shepherd JK Flores T Rodgers RJ Blanchard RJ Caroline Blanchard D. The anxiety/defense test battery: influence of gender and ritanserin treatment on antipredator defensive behavior. Physiol Behav. (1992) 51:277–85. 10.1016/0031-9384(92)90141-N 1557438

[184] Perrot-Sinal TS Gregus A Boudreau D Kalynchuk LE. Sex and repeated restraint stress interact to affect cat odor-induced defensive behavior in adult rats. Brain Res. (2004) 1027:161–72. 10.1016/j.brainres.2004.08.056 15494167

[185] Falconer EM Galea LAM. Sex differences in cell proliferation, cell death and defensive behavior following acute predator odor stress in adult rats. Brain Res. (2003) 975:22–36. 10.1016/S0006-8993(03)02542-3 12763590

[186] Pentkowski NS Litvin Y Blanchard DC Blanchard RJ. Effects of estrus cycle stage on defensive behavior in female Long-Evans hooded rats. Physiol Behav. (2018) 194:41–7. 10.1016/j.physbeh.2018.04.028 29689249

[187] Li XF Adekunbi DA Alobaid HM Li S Pilot M Lightman SL. Role of the posterodorsal medial amygdala in predator odour stress-induced puberty delay in female rats. J Neuroendocrinol. (2019) 31:1–12. 10.1111/jne.12719 30963653 PMC6563483

[188] Vogel JR Beer B Clody DE. A simple and reliable conflict procedure for testing anti-anxiety agents. Psychopharmacologia. (1971) 21:1–7. 10.1007/BF00403989 5105868

[189] Millan MJ. The neurobiology and control of anxious states. Prog Neurobiol. (2003) 70:83–244. 10.1016/S0301-0082(03)00087-X 12927745

[190] Millan MJ Brocco M. The Vogel conflict test: procedural aspects, γ-aminobutyric acid, glutamate and monoamines. Eur J Pharmacol. (2003) 463:67–96. 10.1016/S0014-2999(03)01275-5 12600703

[191] Moreira FA Aguiar DC Guimarães FS. Anxiolytic-like effect of cannabidiol in the rat Vogel conflict test. Prog Neuro-Psychopharmacol Biol Psychiatry. (2006) 30:1466–71. 10.1016/j.pnpbp.2006.06.004 16876926

[192] Sant'Ana AB Vilela-Costa HH Vicente MA Hernandes PM de Andrade TGCS Zangrossi H. Role of 5-HT2C receptors of the dorsal hippocampus in the modulation of anxiety- and panic-related defensive responses in rats. Neuropharmacology. (2019) 148:311–9. 10.1016/j.neuropharm.2019.01.026 30685402

[193] Vicente MA Zangrossi H. Serotonin-2C receptors in the basolateral nucleus of the amygdala mediate the anxiogenic effect of acute imipramine and fluoxetine administration. Int J Neuropsychopharmacol. (2012) 15:389–400. 10.1017/S1461145711000873 21733232

[194] Basso AM Gallagher KB Mikusa JP Rueter LE. Vogel conflict test: sex differences and pharmacological validation of the model. Behav Brain Res. (2011) 218:174–83. 10.1016/j.bbr.2010.11.041 21115068

[195] Peričić D Pivac N. Sex differences in conflict behaviour and in plasma corticosterone levels. J Neural Transm. (1995) 101:213–21. 10.1007/BF01271558 8695051

[196] Peričić D Pivac N. Effects of diazepam on conflict behaviour and on plasma corticosterone levels in male and female rats. Naunyn Schmiedebergs Arch Pharmacol. (1996) 353:369–76. 8935702 10.1007/BF00261432

[197] Barreto-Estrada JL Barreto J Fortis-Santiago Y Rivera-Ramos I Fortis-Santiago A Jorge JC. Modulation of affect after chronic exposure to the anabolic steroid 17α-methyltestosterone in adult mice. Behav Neurosci. (2004) 118:1071–9. 10.1037/0735-7044.118.5.1071 15506889

[198] Curzon P Rustay NR Browman KE. Cued contextual fear conditioning for rodents. In: Buccafusco JJ, editor. Methods of Behavior Analysis in Neuroscience (Boca Raton, FL: CRC Press/Taylor & Francis).

[199] Davis M. Pharmacological and anatomical analysis of fear conditioning. NIDA Res Monogr Ser. (1986) 100:126–62. 10.1037/0735-7044.100.6.814 2247135

[200] De Jongh R Geyer MA Olivier B Groenink L. The effects of sex and neonatal maternal separation on fear-potentiated and light-enhanced startle. Behav Brain Res. (2005) 161:190–6. 10.1016/j.bbr.2005.02.004 15878207

[201] Gresack JE Schafe GE Orr PT Frick KM. Sex differences in contextual fear conditioning are associated with differential ventral hippocampal extracellular signal-regulated kinase activation. Neuroscience. (2009) 159:451–67. 10.1016/j.neuroscience.2009.01.009 19171181

[202] Maren S De Oca B Fanselow MS. Sex differences in hippocampal long-term potentiation (LTP) and Pavlovian fear conditioning in rats: positive correlation between LTP and contextual learning. Brain Res. (1994) 661:25–34. 10.1016/0006-8993(94)91176-2 7834376

[203] Pettersson R Hagsäter SM Eriksson E. Serotonin depletion eliminates sex differences with respect to context-conditioned immobility in rat. Psychopharmacology. (2016) 233:1513–21. 10.1007/s00213-016-4246-5 26905688

[204] Cossio R Carreira MB Vásquez CE Britton GB. Sex differences and estrous cycle effects on foreground contextual fear conditioning. Physiol Behav. (2016) 163:305–11. 10.1016/j.physbeh.2016.05.026 27195460

[205] Maeng LY Milad MR. Sex differences in anxiety disorders: interactions between fear, stress, and gonadal hormones. Horm Behav. (2015) 76:106–17. 10.1016/j.yhbeh.2015.04.002 25888456 PMC4823998

[206] Milad MR Igoe SA Lebron-Milad K Novales JE. Estrous cycle phase and gonadal hormones influence conditioned fear extinction. Neuroscience. (2009) 164:887–95. 10.1016/j.neuroscience.2009.09.011 19761818 PMC2783784

[207] Pryce CR Lehmann J Feldon J. Effect of sex on fear conditioning is similar for context and discrete CS in Wistar, Lewis and Fischer rat strains. Pharmacol Biochem Behav. (1999) 64:753–9. 10.1016/S0091-3057(99)00147-1 10593198

[208] Caldwell KK Solomon ER Smoake JJW Djatche de Kamgaing CD Allan AM. Sex-specific deficits in biochemical but not behavioral responses to delay fear conditioning in prenatal alcohol exposure mice. Neurobiol Learn Mem. (2018) 156:1–16. 10.1016/j.nlm.2018.10.002 30316893 PMC6261673

[209] Keiser AA Turnbull LM Darian MA Feldman DE Song I Tronson NC. Sex differences in context fear generalization and recruitment of hippocampus and amygdala during retrieval. Neuropsychopharmacology. (2017) 42:397–407. 10.1038/npp.2016.174 27577601 PMC5399239

[210] Baran SE Armstrong CE Niren DC Hanna JJ Conrad CD. Chronic stress and sex differences on the recall of fear conditioning and extinction. Neurobiol Learn Mem. (2009) 91:323–32. 10.1016/j.nlm.2008.11.005 19073269 PMC2673234

[211] Borkar CD Dorofeikova M Le QSE Vutukuri R Vo C Hereford D. Sex differences in behavioral responses during a conditioned flight paradigm. Behav Brain Res. (2020) 389:112623. 10.1016/j.bbr.2020.112623 32348872

[212] Carvalho MC Genaro K Leite-Panissi CRA Lovick TA. Influence of estrous cycle stage on acquisition and expression of fear conditioning in female rats. Physiol Behav. (2021) 234:113372. 10.1016/j.physbeh.2021.113372 33647267

[213] Blume SR Freedberg M Vantrease JE Chan R Padival M Record MJ. Sex- And estrus-dependent differences in rat basolateral amygdala. J Neurosci. (2017) 37:10567–86. 10.1523/JNEUROSCI.0758-17.2017 28954870 PMC5666581

[214] Adkins JM Lynch JF Hagerdorn P Esterhuizen M Jasnow AM. Anterior cingulate cortex and dorsal hippocampal glutamate receptors mediate generalized fear in female rats. Psychoneuroendocrinology. (2019) 107:109–18. 10.1016/j.psyneuen.2019.05.009 31125757 PMC7779207

[215] Adkins JM Lynch J Gray M Jasnow AM. Presynaptic GABAB receptor inhibition sex dependently enhances fear extinction and attenuates fear renewal. Psychopharmacology. (2021) 238:2059–71. 10.1007/s00213-021-05831-w 33855580 PMC8295214

[216] Lynch JF Winiecki P Vanderhoof T Riccio DC Jasnow AM. Hippocampal cytosolic estrogen receptors regulate fear generalization in females. Neurobiol Learn Mem. (2016) 130:83–92. 10.1016/j.nlm.2016.01.010 26851128

[217] Kreutzmann JC Fendt M. Intranasal oxytocin compensates for estrus cycle-specific reduction of conditioned safety memory in rats: implications for psychiatric disorders. Neurobiol Stress. (2021) 14:100313. 10.1016/j.ynstr.2021.100313 33778132 PMC7985696

[218] Craske MG Hermans D Vervliet B. State-of-the-art and future directions for extinction as a translational model for fear and anxiety. Philos Trans R Soc B Biol Sci. (2018) 373:20180432. 10.1098/rstb.2018.0432 29352025 PMC5790824

[219] Pittig A Treanor M LeBeau RT Craske MG. The role of associative fear and avoidance learning in anxiety disorders: gaps and directions for future research. Neurosci Biobehav Rev. (2018) 88:117–40. 10.1016/j.neubiorev.2018.03.015 29550209

[220] Duits P Cath DC Lissek S Hox JJ Hamm AO Engelhard IM. Updated meta-analysis of classical fear conditioning in the anxiety disorders. Depress Anxiety. (2015) 32:239–53. 10.1002/da.22353 25703487

[221] Milad MR Pitman RK Ellis CB Gold AL Shin LM Lasko NB. Neurobiological basis of failure to recall extinction memory in posttraumatic stress disorder. Biol Psychiatry. (2009) 66:1075–82. 10.1016/j.biopsych.2009.06.026 19748076 PMC2787650

[222] Singewald N Holmes A. Rodent models of impaired fear extinction. Psychopharmacology. (2019) 236:21–32. 10.1007/s00213-018-5054-x 30377749 PMC6373188

[223] Breslau N. Gender differences in trauma and posttraumatic stress disorder. Ital J Gender-Specific Med. (2002) 5:34–40. 11859685

[224] Zeidan MA Igoe SA Linnman C Vitalo A Levine JB Klibanski A. Estradiol modulates medial prefrontal cortex and amygdala activity during fear extinction in women and female rats. Biol Psychiatry. (2011) 70:920–7. 10.1016/j.biopsych.2011.05.016 21762880 PMC3197763

[225] Urien L Stein N Ryckman A Bell L Bauer EP. Extended amygdala circuits are differentially activated by context fear conditioning in male and female rats. Neurobiol Learn Mem. (2021) 180:107401. 10.1016/j.nlm.2021.107401 33581315 PMC8076097

[226] Morena M Nastase AS Santori A Cravatt BF Shansky RM Hill MN. Sex-dependent effects of endocannabinoid modulation of conditioned fear extinction in rats. Br J Pharmacol. (2021) 178:983–96. 10.1111/bph.15341 33314038 PMC8311789

[227] Pellman BA Schuessler BP Tellakat M Kim JJ. Cognition and behavior sexually dimorphic risk mitigation strategies in rats. (2017). 4:ENEURO.0288–16.2017. 10.1523/ENEURO.0288-16.2017 28197550 PMC5292597

[228] Pellman BA Kim JJ. What can ethobehavioral studies tell us about the brain's fear system? Trends Neurosci. (2016) 39:420–31. 10.1016/j.tins.2016.04.001 27130660 PMC4884474

[229] Simpson J Ryan C Curley A Mulcaire J Kelly JP. Sex differences in baseline and drug-induced behavioural responses in classical behavioural tests. Prog Neuro-Psychopharmacology Biol Psychiatry. (2012) 37:227–36. 10.1016/j.pnpbp.2012.02.004 22353173

[230] Kim HS Sakai N Saito K Fujita S Ishizuka M. Diazepam metabolism in the kidneys of male and female rats of various strains. J Vet Med Sci. (2010) 72:7–11. 10.1292/jvms.09-0127 19893285

[231] Wilson MA Burghardt PR Ford KA Wilkinson MB Primeaux SD. Anxiolytic effects of diazepam and ethanol in two behavioral models: comparison of males and females. Pharmacol Biochem Behav. (2004) 78:445–58. 10.1016/j.pbb.2004.04.017 15251253

[232] Fernández-Guasti A Picazo O. Anxiolytic actions of diazepam, but not of buspirone, are influenced by gender and the endocrine stage. Behav Brain Res. (1997) 88:213–8. 10.1016/S0166-4328(97)00047-8 9404630

[233] Carey MP Billing AE Fry JP. Fluctuations in responses to diazepam during the oestrous cycle in the mouse. Pharmacol Biochem Behav. (1992) 41:719–25. 10.1016/0091-3057(92)90218-5 1594639

[234] Fernández-Guasti A Picazo O. The actions of diazepam and serotonergic anxiolytics vary according to the gender and the estrous cycle phase. Pharmacol Biochem Behav. (1990) 37:77–81. 10.1016/0091-3057(90)90044-I 1979876

[235] Nomikos GG Spyraki C. Influence of oestrogen on spontaneous and diazepam-induced exploration of rats in an elevated plus maze. Neuropharmacology. (1988) 27:691–6. 10.1016/0028-3908(88)90077-9 3419550

[236] Martin JV Williams DB. Benzodiazepine binding varies with stage of estrous cycle in unwashed membranes from mouse brain. Life Sci. (1995) 57:1903–9. 10.1016/0024-3205(95)02177-k 7475940

[237] Monleón S Urquiza A Carmen Arenas M Vinader-Caerols C Parra A. Chronic administration of fluoxetine impairs inhibitory avoidance in male but not female mice. Behav Brain Res. (2002) 136:483–8. 10.1016/S0166-4328(02)00194-8 12429411

[238] Pinna G Costa E Guidotti A. SSRIs act as selective brain steroidogenic stimulants (SBSSs) at low doses that are inactive on 5-HT reuptake. Curr Opin Pharmacol. (2009) 9:24–30. 10.1016/j.coph.2008.12.006 19157982 PMC2670606

[239] Gruene TM Flick K Stefano A Shea SD Shansky RM. Sexually divergent expression of active and passive conditioned fear responses in rats. Elife. (2015) 4:e11352. 10.7554/eLife.11352 26568307 PMC4709260

[240] Colom-Lapetina J Li AJ Pelegrina-Perez TC Shansky RM. Behavioral diversity across classic rodent models is sex-dependent. Front Behav Neurosci. (2019) 13:45. 10.3389/fnbeh.2019.00045 30894806 PMC6414415

[241] Mobbs D Adolphs R Fanselow MS Barrett LF LeDoux JE Ressler K. Viewpoints: approaches to defining and investigating fear. Nat Neurosci. (2019) 22:1205–16. 10.1038/s41593-019-0456-6 31332374 PMC6943931

[242] Würbel H. Ideal homes? Housing effects on rodent brain and behaviour. Trends Neurosci. (2001) 24:207–11. 10.1016/S0166-2236(00)01718-5 11250003

[243] Mobbs D Petrovic P Marchant JL Hassabis D Weiskopf N. When fear is near : *Science.* (2007) 1119:1079–83. 10.1126/science.1144298 17717184 PMC2648508

[244] Jolles JW Boogert NJ van den Bos R. Sex differences in risk-taking and associative learning in rats. R Soc Open Sci. (2015) 2:150485. 10.1098/rsos.150485 26716004 PMC4680619

[245] Hantsoo L Epperson CN. Allopregnanolone in premenstrual dysphoric disorder (PMDD): evidence for dysregulated sensitivity to GABA-A receptor modulating neuroactive steroids across the menstrual cycle. Neurobiol Stress. (2020) 12:100213. 10.1016/j.ynstr.2020.100213 32435664 PMC7231988

[246] Nillni YI Rasmusson AM Paul EL Pineles SL. The impact of the menstrual cycle and underlying hormones in anxiety and PTSD: what do we know and where do we go from here? Curr Psychiatry Rep. (2021) 23:8. 10.1007/s11920-020-01221-9 33404887 PMC8819663

[247] Sundström-Poromaa I. The menstrual cycle influences emotion but has limited effect on cognitive function. Vitam Horm. (2018) 107:349–76. 10.1016/bs.vh.2018.01.016 29544637

